# benchdamic: benchmarking of differential abundance methods for microbiome data

**DOI:** 10.1093/bioinformatics/btac778

**Published:** 2022-12-07

**Authors:** Matteo Calgaro, Chiara Romualdi, Davide Risso, Nicola Vitulo

**Affiliations:** Department of Biotechnology, University of Verona, Verona 37134, Italy; Department of Biology, University of Padova, Padova 35131, Italy; Department of Statistical Sciences, University of Padova, Padova 35121, Italy; Department of Biotechnology, University of Verona, Verona 37134, Italy

## Abstract

**Summary:**

Recently, an increasing number of methodological approaches have been proposed to tackle the complexity of metagenomics and microbiome data. In this scenario, reproducibility and replicability have become two critical issues, and the development of computational frameworks for the comparative evaluations of such methods is of utmost importance. Here, we present benchdamic, a Bioconductor package to benchmark methods for the identification of differentially abundant taxa.

**Availability and implementation:**

benchdamic is available as an open-source R package through the Bioconductor project at https://bioconductor.org/packages/benchdamic/.

**Supplementary information:**

Supplementary data are available at *Bioinformatics* online.

## 1 Introduction

Differential abundance (DA) analysis identifies significant differences in the microbial community composition between groups of samples, providing new insights into the composition of microbial communities and on their associations with the environment. Although many approaches have been proposed for DA analysis, it is widely recognized that the best method (i.e. a method with performances uniformly better than all the others) does not exist and that a careful exploratory data analysis is necessary to address methodological choices (Calgaro *et al.*, 2020; Hawinkel *et al.*, 2019; Nearing *et al.*, 2022; Thorsen *et al.*, 2016; Weiss *et al.*, 2017).

Building on our previous work (Calgaro *et al.*, 2020), we present the benchdamic R/Bioconductor package, which provides a computational framework to guide researchers in the selection of the method that best fits their data.

**Fig. 1.**
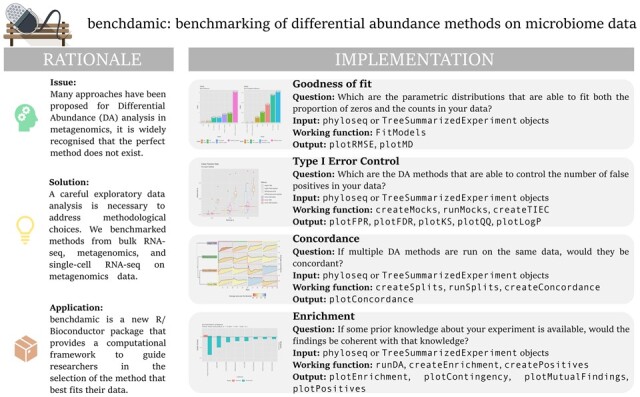
Graphical abstract. Each box on the right represents a step of the analysis where information about the research question, type of input data, working functions and outputs are reported

The structure of benchdamic can be summarized into four main parts (Fig. 1). Each section is developed to answer specific questions when comparing samples from different experimental groups, namely (i) the ability for a given statistical distribution to successfully fit the input data, with particular focus on sparsity and their count nature; (ii) the ability of the DA methods to control the type I error; (iii) the concordance among methods; and (iv) the accuracy of the findings based on a priori biological knowledge. Altogether, benchdamic is a flexible and customizable framework that can be used for the benchmarking of new and existing DA methods.

## 2 Implementation

benchdamic builds on existing R/Bioconductor infrastructure packages: the primary input of benchdamic’s main functions is a phyloseq or a TreeSummarizedExperiment object (Huang *et al.*, 2021; McMurdie and Holmes, 2013). Ready-to-use normalization and DA methods included in benchdamic are based on the edgeR (Robinson *et al.*, 2010), DESeq2 (Love *et al.*, 2014), limma-voom (Law *et al.*, 2014; Phipson *et al.*, 2016; Ritchie *et al.*, 2015), metagenomeSeq (Paulson *et al.*, 2013), ALDEx2 (Fernandes *et al.*, 2014, 2013), corncob (Martin *et al.*, 2020), MAST (Finak *et al.*, 2015), Seurat (Butler *et al.*, 2018), dearseq (Gauthier *et al.*, 2020), NOISeq (Tarazona *et al.*, 2015), ANCOMBC (Lin and Peddada, 2020; Mandal *et al.*, 2015) and zinbwave (Risso *et al.*, 2018; Van den Berge *et al.*, 2018) packages. Combinations of parameters are possible as well as the inclusion of custom methods (see Supplementary material Section S3).

In the following sections, we briefly outline the main functionality of the package. See Calgaro *et al.* (2020) for technical details on how these metrics are computed.

### 2.1 Goodness of fit

DA statistical models are based on different statistical distributions. Five different distributions are available in benchdamic for testing the goodness of fit on user-provided data: negative binomial, zero-inflated negative binomial, zero-inflated Gaussian, truncated Gaussian and Dirichlet-multinomial (see Supplementary material Section S2). Goodness of fit is measured by the ability of each method to correctly estimate the average counts and the probability of observing a zero.

### 2.2 Type I error control

To investigate the Type I error rate control of each DA method (i.e. the probability of the statistical test to call a feature DA when it is not) mock datasets with no true DA are generated starting from the user-provided data (see Supplementary material Section S4).

Briefly, the dataset is split into two random subsets and DA analysis, based on a chosen list of methods, is performed. The process is repeated *N* times (*N *≥ 1000 suggested). The performances of each method are then summarized and graphically represented considering the false positive rate, false discovery rate, and departure from uniformity for the *P*-values distribution.

### 2.3 Concordance

benchdamic can be used to measure the between-method concordance (BMC), in which a DA method is compared to other methods in the same dataset, and the within-method concordance (WMC), in which a method is compared to itself in two random subsets of the same dataset (Supplementary material Section S5). Firstly, the dataset is randomly divided in half to obtain two subsets (Subset1 and Subset2) with samples from two or more biological groups, then DA analysis is performed between two groups, independently on each subset. The process is repeated *N* times (*N* ≥ 100 suggested) and average WMC and BMC metrics are computed and summarized using a heatmap representation.

### 2.4 Enrichment

Enrichment analysis can provide an alternative way of ranking methods in terms of their ability to identify, as significantly different, taxa that are known to be differentially abundant between two groups. DA analysis needs to be performed on a dataset where some a priori knowledge is available (Supplementary material Section S6). Given the direction of the DA features (over- or under-abundant) and the expected group in which the features should be differentially abundant according to the prior knowledge, several contingency tables are created for each DA method. A Fisher exact test is then performed to test the enrichment and the DA features identified by more than one method are highlighted. Additionally, the users will be able to rank the methods based on the difference between the total number of true positives and false positives for several thresholds (based on *P*-values, adjusted *P*-values or other statistics). The same approach can also be used to perform power analysis using simulated data (Supplementary material Section S6.8).

## 3 Conclusions

The benchdamic R/Bioconductor package aims to be a support tool for the identification of DA microbial taxa and the benchmarking of new methods. We envision two main uses of our package: (i) for practitioners interested in performing DA analysis on a new dataset, benchdamic can be used to identify the best DA methods among those already in the literature; (ii) for method developers interested in benchmarking their new approach, benchdamic can be used as an impartial tool to evaluate the relative merits of the new method compared to what is already available.

benchdamic is available as an open-source package through the Bioconductor project. The package includes a vignette with a detailed tutorial.

The future of benchdamic is oriented to the addition of new aspects of analysis e.g. new normalization methods and new DA approaches.

## Supplementary Material

btac778_Supplementary_Data

## References

[btac778-B1] Butler A. et al (2018) Integrating single-cell transcriptomic data across different conditions, technologies, and species. Nat. Biotechnol., 36, 411–420. 29608179 10.1038/nbt.4096 PMC6700744

[btac778-B2] Calgaro M. et al (2020) Assessment of statistical methods from single cell, bulk RNA-seq, and metagenomics applied to microbiome data. Genome Biol., 21, 191. 32746888 10.1186/s13059-020-02104-1 PMC7398076

[btac778-B3] Fernandes A.D. et al (2013) ANOVA-Like differential expression (ALDEx) analysis for mixed population RNA-Seq. PLoS One, 8, e67019. 23843979 10.1371/journal.pone.0067019 PMC3699591

[btac778-B4] Fernandes A.D. et al (2014) Unifying the analysis of high-throughput sequencing datasets: characterizing RNA-seq, 16S rRNA gene sequencing and selective growth experiments by compositional data analysis. Microbiome, 2, 15. 24910773 10.1186/2049-2618-2-15 PMC4030730

[btac778-B5] Finak G. et al (2015) MAST: a flexible statistical framework for assessing transcriptional changes and characterizing heterogeneity in single-cell RNA sequencing data. Genome Biol., 16, 278. 26653891 10.1186/s13059-015-0844-5 PMC4676162

[btac778-B6] Gauthier M. et al (2020) Dearseq: a variance component score test for RNA-seq differential analysis that effectively controls the false discovery rate. NAR Genom. Bioinform., 2, lqaa093. 33575637 10.1093/nargab/lqaa093 PMC7676475

[btac778-B7] Hawinkel S. et al (2019) A broken promise: microbiome differential abundance methods do not control the false discovery rate. Brief Bioinform., 20, 210–221. 28968702 10.1093/bib/bbx104

[btac778-B8] Huang R. et al (2021) TreeSummarizedExperiment: a S4 class for data with hierarchical structure. F1000Research, 9, 1246. 10.12688/f1000research.26669.1 PMC7684683 33274053

[btac778-B9] Law C.W. et al (2014) Voom: precision weights unlock linear model analysis tools for RNA-seq read counts. Genome Biol., 15, R29. 24485249 10.1186/gb-2014-15-2-r29 PMC4053721

[btac778-B10] Lin H. , Peddada S.D. (2020) Analysis of compositions of microbiomes with bias correction. Nat. Commun., 11, 3514. 32665548 10.1038/s41467-020-17041-7 PMC7360769

[btac778-B11] Love M.I. et al (2014) Moderated estimation of fold change and dispersion for RNA-seq data with DESeq2. Genome Biol., 15, 550. 25516281 10.1186/s13059-014-0550-8 PMC4302049

[btac778-B12] Mandal S. et al (2015) Analysis of composition of microbiomes: a novel method for studying microbial composition. Microb. Ecol. Health Dis., 26, 27663. 26028277 10.3402/mehd.v26.27663 PMC4450248

[btac778-B13] Martin B.D. et al (2020) Modeling microbial abundances and dysbiosis with beta-binomial regression. Ann. Appl. Stat., 14, 94–115. 32983313 10.1214/19-aoas1283 PMC7514055

[btac778-B14] McMurdie P.J. , Holmes S. (2013) Phyloseq: an R package for reproducible interactive analysis and graphics of microbiome census data. PLoS One, 8, e61217. 23630581 10.1371/journal.pone.0061217 PMC3632530

[btac778-B15] Nearing J.T. et al (2022) Microbiome differential abundance methods produce different results across 38 datasets. Nat. Commun., 13, 342. 35039521 10.1038/s41467-022-28034-z PMC8763921

[btac778-B16] Paulson J.N. et al (2013) Differential abundance analysis for microbial marker-gene surveys. Nat. Methods, 10, 1200–1202. 24076764 10.1038/nmeth.2658 PMC4010126

[btac778-B17] Phipson B. et al (2016) Robust hyperparameter estimation protects against hypervariable genes and improves power to detect differential expression. Ann. Appl. Stat., 10, 946–963. 28367255 10.1214/16-AOAS920 PMC5373812

[btac778-B18] Risso D. et al (2018) A general and flexible method for signal extraction from single-cell RNA-seq data. Nat. Commun., 9, 284. 29348443 10.1038/s41467-017-02554-5 PMC5773593

[btac778-B19] Ritchie M.E. et al (2015) Limma powers differential expression analyses for RNA-sequencing and microarray studies. Nucleic Acids Res., 43, e47. 25605792 10.1093/nar/gkv007 PMC4402510

[btac778-B20] Robinson M.D. et al (2010) edgeR: a bioconductor package for differential expression analysis of digital gene expression data. Bioinformatics, 26, 139–140. 19910308 10.1093/bioinformatics/btp616 PMC2796818

[btac778-B21] Tarazona S. et al (2015) Data quality aware analysis of differential expression in RNA-seq with NOISeq R/bioc package. Nucleic Acids Res., **43**, e140. 10.1093/nar/gkv711 PMC4666377 26184878

[btac778-B22] Thorsen J. et al (2016) Large-scale benchmarking reveals false discoveries and count transformation sensitivity in 16S rRNA gene amplicon data analysis methods used in microbiome studies. Microbiome, 4, 62. 27884206 10.1186/s40168-016-0208-8 PMC5123278

[btac778-B23] Van den Berge K. et al (2018) Observation weights unlock bulk RNA-seq tools for zero inflation and single-cell applications. Genome Biol., 19, 24. 29478411 10.1186/s13059-018-1406-4 PMC6251479

[btac778-B24] Weiss S. et al (2017) Normalization and microbial differential abundance strategies depend upon data characteristics. Microbiome, 5, 27. 28253908 10.1186/s40168-017-0237-y PMC5335496

