# Supplementary material for: benchdamic: benchmarking of differential abundance methods for microbiome data
Source: Bioinformatics. 2022 Dec 7;39(1):btac778. doi: 10.1093/bioinformatics/btac778 (PMC9825737; doi:10.1093/bioinformatics/btac778)
Supplement: btac778_Supplementary_Data [file btac778_supplementary_data.pdf]

benchdamic: benchmarking of differential abundance method for microbiome data

supplementary material

Matteo Calgaro Chiara Romualdi Davide Risso Nicola Vitulo

Contents

1 Introduction 3
1.1 Installation . . . 3
1.2 Data loading . . . 3

2 Goodness of Fit 5
2.1 GOF structure . . . 5
2.2 Parametric distributions . . . 5
2.3 Comparing estimated and observed values . . . 7
2.4 Visualization . . . 9
2.5 Discussion about GOF . . . 13

3 DA methods 14
3.1 Add a custom DA method . . . 16

4 Type I Error Control 17
4.1 TIEC structure . . . 17
4.2 Create mock comparisons . . . 18
4.3 Set up normalizations and DA methods . . . 18
4.4 Counting the False Positives . . . 24
4.5 Visualization . . . 25
4.6 Discussion about TIEC . . . 32

5 Concordance 33
5.1 Concordance structure . . . 33
5.2 Split datasets . . . 33
5.3 Set up normalizations and DA methods . . . 34
5.4 Comparing the concordances . . . 37
5.5 Visualization . . . 39
5.6 Discussion about Concordance . . . 41

6 Enrichment analysis 42
6.1 Enrichment structure . . . 42
6.2 *A priori* knowledge . . . 42
6.3 Set up normalizations and DA methods . . . 43
6.4 Testing the enrichment . . . 44
6.5 Visualization . . . 45
6.6 True and False Positives . . . 48
6.7 Enrichment without direction . . . 49
6.8 Enrichment analysis for simulated data . . . 52

6.9 Discussion about Enrichment 58

7 Session Info **61**

References **65**

1 Introduction

This document is a static version of the vignette for R/Bioconductor package `benchdamic`. It provides an introductory example on how to work with the analysis framework firstly proposed in (Calgaro *et al.*, 2020).

Some methods for differential abundance (DA) analysis are tested on microbiome datasets. Performances of each method are evaluated with respect to i) suitability of distributional assumptions (GOF), ii) ability to control false positives (TIEC), iii) concordance of the findings, and iv) enrichment of DA microbial species in specific conditions.

1.1 Installation

To install this package, start R (version "4.2") and enter:

```
if (!require("BiocManager", quietly = TRUE))
    install.packages("BiocManager")

BiocManager::install("benchdamic")
```

or use:

```
if (!require("devtools", quietly = TRUE))
    install.packages("devtools")

devtools::install_github("mcalgaro93/benchdamic")
```

Then, load some packages for basic functions and data:

```
library(benchdamic)
# Parallel computation
library(BiocParallel)
# Generate simulated data
library(SPsimSeq)
# Data management
library(phyloseq)
library(SummarizedExperiment)
library(plyr)
# Graphics and tables
library(ggplot2)
library(cowplot)
library(kableExtra)
```

1.2 Data loading

All datasets used in `benchdamic` are downloaded using the `HMP16SData` Bioconductor package (Schiffer *et al.*, 2019).

For GOF and TIEC analyses a homogeneous group of samples (*e.g.*, only samples from a specific experimental condition, phenotype, treatment, body site, etc.) *ps_stool_16S* is used (use `help("ps_stool_16S")` for details). It contains 16S data from:

- 32 stool samples from participants of the Human Microbiome Project;
- 71 taxa, all features having the same genus-level taxonomic classification are collapsed together (a total of 71 taxa corresponding to 71 genera).

```
data("ps_stool_16S")
ps_stool_16S
```

```
## phyloseq-class experiment-level object
## otu_table()   OTU Table:         [ 71 taxa and 32 samples ]
## sample_data() Sample Data:       [ 32 samples by 7 sample variables ]
## tax_table()   Taxonomy Table:    [ 71 taxa by 6 taxonomic ranks ]
## phy_tree()    Phylogenetic Tree: [ 71 tips and 70 internal nodes ]
```

For Concordance and Enrichment analyses the *ps_plaque_16S* dataset is used (use `help("ps_plaque_16S")` for details). It contains 16S data from:

- 30 participants of the Human Microbiome Project;
- samples collected from subgingival plaque and supragingival plaque for each subject (a total of 60 samples);
- 88 taxa, all features having the same genus-level taxonomic classification are collapsed together (a total of 88 taxa corresponding to 88 genera).

```
data("ps_plaque_16S")
ps_plaque_16S
```

```
## phyloseq-class experiment-level object
## otu_table()   OTU Table:         [ 88 taxa and 60 samples ]
## sample_data() Sample Data:       [ 60 samples by 7 sample variables ]
## tax_table()   Taxonomy Table:    [ 88 taxa by 6 taxonomic ranks ]
## phy_tree()    Phylogenetic Tree: [ 88 tips and 87 internal nodes ]
```

2 Goodness of Fit

Assumption: Many DA detection methods are based on parametric distributions.

Research question: Which are the parametric distributions that can fit both the proportion of zeros and the counts in your data?

2.1 GOF structure

As different methods rely on different statistical distributions to perform DA analysis, the goodness of fit (GOF) of the statistical models underlying some of the DA methods on a 16S dataset is assessed. For each model, its ability to correctly estimate the average counts and the proportion of zeroes by taxon is evaluated.

Five distributions are considered: (1) the negative binomial (**NB**) used in `edgeR` and `DeSeq2` (Robinson *et al.*, 2010; Love *et al.*, 2014), (2) the zero-inflated negative binomial (**ZINB**) used in ZINB-WaVE (Risso *et al.*, 2018), (3) the truncated Gaussian Hurdle model of `MAST` (Finak *et al.*, 2015), (4) the zero-inflated Gaussian (**ZIG**) mixture model of `metagenomeSeq` (Paulson *et al.*, 2013), and (5) the Dirichlet-Multinomial (**DM**) distribution underlying `ALDEx2` Monte-Carlo sampling (Fernandes *et al.*, 2014) and multivariate extension of the beta-binomial distribution used by `conrcob` (Martin *et al.*, 2020).

The relationships between the functions used in this section are explained by the diagram in Figure 1. To help with the reading: green boxes represent the inputs or the outputs, red boxes are the methods and blue boxes are the main parameters of those method.

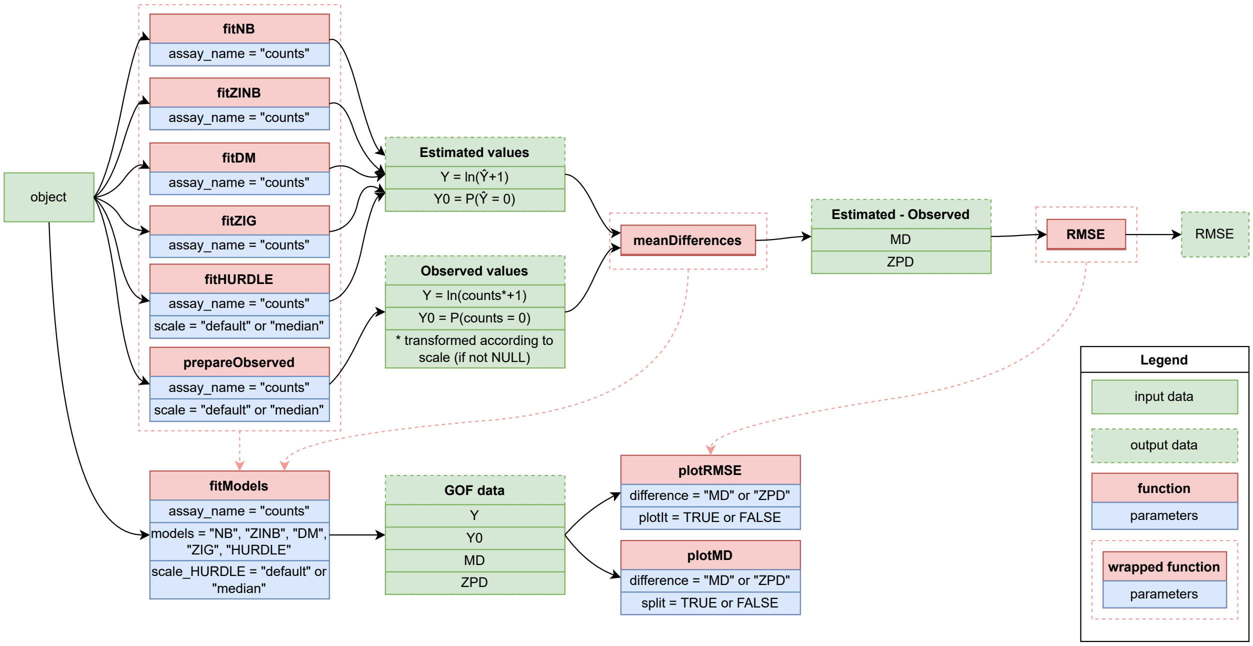

Figure 1: Goodness of Fit diagram.

2.2 Parametric distributions

2.2.1 Negative Binomial and Zero-Inflated Negative Binomial Models

For any $\mu \geq 0$ and $\theta > 0$, let $f_{NB}(\cdot;\mu,\theta)$ denote the probability mass function (PMF) of the negative binomial (NB) distribution with mean μ and inverse dispersion parameter θ, namely:

$$f_{NB} = \frac{\Gamma(y+\theta)}{\Gamma(y+1)\Gamma(\theta)} \left(\frac{\theta}{\theta+1}\right)^{\theta} \left(\frac{\mu}{\mu+\theta}\right)^{y}, \forall y \in \mathbb{N}$$

Note that another parametrization of the NB PMF is in terms of the dispersion parameter $\psi = \theta^{-1}$ (although θ is also sometimes called dispersion parameter in the literature). In both cases, the mean of the NB distribution is μ and its variance is:

$$\sigma^2 = \mu + \frac{\mu^2}{\theta} = \mu + \psi\mu^2$$

In particular, the NB distribution boils down to a Poisson distribution when $\psi = 0 \iff \theta = +\infty$.

For any $\pi \in [0, 1]$, let $f_{ZINB}(\cdot; \mu, \theta, \pi)$ be the PMF of the ZINB distribution given by:

$$f_{ZINB}(\cdot; \mu, \theta, \pi) = \pi\delta_0(y) + (1 - \pi)f_{NB}(y; \mu, \theta), \forall y \in \mathbb{N}$$

where $\delta_0(\cdot)$ is the Dirac function. Here, π can be interpreted as the probability that a 0 is observed instead of the actual count, resulting in an inflation of zeros compared to the NB distribution, hence the name ZINB.

To fit these distributions on real count data the `edgeR` (Robinson *et al.*, 2010) and `zinbwave` (Risso *et al.*, 2018) packages are used. In `benchdamic` they are implemented in the `fitNB()` and `fitZINB()` functions.

2.2.2 Zero-Inflated Gaussian Model

The raw count for sample j and feature i is denoted by c_{ij}. The zero-inflated model is defined for the continuity-corrected logarithm of the raw count data: $y_{ij} = log_2(c_{ij} + 1)$ as a mixture of a point mass at zero $I_0(y)$ and a count distribution $f_{count}(y; \mu, \sigma^2) \sim N(\mu, \sigma^2)$. Given mixture parameters π_j, we have that the density of the ZIG distribution for feature i, in sample j with s_j total counts is:

$$f_{ZIG}(y_{ij}; s_j, \beta, \mu_i, \sigma_i^2) = \pi_j(s_j) \cdot I_0(y_{ij}) + (1 - \pi_j(s_j)) \cdot f_{count}(y_{ij}; \mu, \sigma^2)$$

The mean model is specified as:

$$E(y_{ij}) = \pi_j + (1 - \pi_j) \cdot \left(b_{i0} + \eta_i log_2\left(\frac{s_j^{\hat{l}}}{N} + 1\right)\right)$$

In this case, parameter b_{i0} is the intercept of the model while the term including the logged normalization factor $log_2\left(\frac{s_j^{\hat{l}}}{N} + 1\right)$ captures feature-specific normalization factors through parameter η_i. In details, $s_j^{\hat{l}}$ is the median scaling factor resulted from the Cumulative Sum Scaling (CSS) normalization procedure. N is a constant fixed by default at 1000 but it should be a number close to the scaling factors to be used as a reference, for this reason a good choice could be the median of the scaling factors (which is used instead of 1000). The mixture parameters $\pi_j(s_j)$ are modeled as a binomial process:

$$log\frac{\pi_j}{1 - \pi_j} = \beta_0 + \beta_1 \cdot log(s_j)$$

To fit this distribution on real count data the `metagenomeSeq` package (Paulson *et al.*, 2013) is used. In `benchdamic` it is implemented in the `fitZIG()` function.

2.2.3 Truncated Gaussian Hurdle Model

The original field of application of this method was the single-cell RNAseq data, where $y = log_2(TPM + 1)$ expression matrix was modeled as a two-part generalized regression model (Finak *et al.*, 2015). In microbiome data that starting point translates to a $y_{ij} = log_2\left(counts_{ij} \cdot \frac{10^6}{libSize_j} + 1\right)$ or a $log_2\left(counts_{ij} \cdot \frac{median(libSize)}{libSize_j} + 1\right)$.

The taxon presence rate is modeled using logistic regression and, conditioning on a sample with the taxon, the transformed abundance level is modeled as Gaussian.

Given normalized, possibly thresholded, abundance y_{ij}, the rate of presence and the level of abundance for the samples were the taxon is present, are modeled conditionally independent for each gene i. Define the indicator z_{ij}, indicating whether taxon i is expressed in sample j (*i.e.*, $z_{ij} = 0$ if $y_{ij} = 0$ and $z_{ij} = 1$ if $y_{ij} > 0$). We fit logistic regression models for the discrete variable Z and a Gaussian linear model for the continuous variable $(Y|Z = 1)$ independently, as follows:

$$logit(Pr(Z_{ij} = 1)) = X_j \beta_i^D$$

$$P(Y_{ij} = y|Z_{ij} = 1) \sim N(X_j \beta_i^C, \sigma_i^2)$$

To estimate this distribution on real count data the `MAST` package (Finak *et al.*, 2015) is used. In `benchdamic` it is implemented in the `fitHURDLE()` function.

2.2.4 Dirichlet-Multinomial Mixture Model

The probability mass function of a n dimensional multinomial sample $y = (y_1, ..., y_n)^T$ with library size $libSize = \sum_{i=1}^{n} y_i$ and parameter $p = (p_1, ..., p_n)$ is:

$$f(y; p) = \binom{libSize}{y} \prod_{i=1}^{n} p_i^{y_i}$$

The mean-variance structure of the MN model doesn't allow over-dispersion, which is common in real data. DM distribution models the probability parameter p in the MN model by a Dirichlet distribution. The probability mass of a n-category count vector y over $libSize$ trials under DM with parameter $\alpha = (\alpha_1, ..., \alpha_n)$, $a_i > 0$ and proportion vector $p \in \Delta_n = \{(p_1, ..., p_n) : p_i \geq 0, \sum_i p_i = 1\}$ is:

$$f(y|\alpha) = \binom{libSize}{y} \frac{\prod_{i=1}^{n} (a_i) y_i}{(\sum_i \alpha_i) \cdot libSize}$$

The mean value for the ith taxon and j^{th} sample of the count matrix is given by $libSize_j \cdot \frac{\alpha_{ij}}{\sum_i a_{ij}}$.

To estimate this distribution on real count data the `MGLM` package (Zhang *et al.*, 2017; Zhang and Zhou, 2022) is used. In `benchdamic` it is implemented in the `fitDM()` function.

2.3 Comparing estimated and observed values

The goodness of fit for the previously described distributions is assessed comparing estimated and observed values. For each taxon the following measures are compared:

- the Mean Difference (MD) *i.e.* the difference between the estimated mean and the observed mean abundance (log scale);
- the Zero Probability Difference (ZPD) *i.e.* the difference between the probability to observe a zero and the observed proportion of samples which have zero counts.

To easily compare estimated and observed mean values the natural logarithm transformation, with the continuity correction ($log(counts + 1)$), is well suited, indeed it reduces the count range making the differences more stable.

Except for the `fitHURDLE()` function, which performs a CPM transformation on the counts (or the one with the median library size), and the `fitZIG()` function which models the $log_2(counts + 1)$, the other methods, `fitNB()`, `fitZINB()`, and `fitDM()`, model the *counts* directly. For these reasons, `fitHURDLE()`'s output should not be compared directly to the observed $log(counts + 1)$ mean values as for the other methods. Instead, the logarithm of the observed CPM (or the one with the median library size) should be used.

Here an example on how to fit a Truncated Gaussian hurdle model:

```
example_HURDLE <- fitHURDLE(
    object = ps_stool_16S,
    scale = "median"
)
head(example_HURDLE)
```

```
##                          Y          Y0
## OTU_97.192   2.152592 0.312760011
## OTU_97.1666        NA 0.967681879
## OTU_97.31213 4.057621 0.094509772
## OTU_97.39094 1.123198 0.749635900
## OTU_97.40451 7.327534 0.001949454
## OTU_97.19587 4.716914 0.063341989
```

The values above are those estimated by the `fitHURDLE()` function. Some *NA* values could be present due to taxa sparsity. The internally used function to prepare for the comparisons the observed counts is `prepareObserved()`, specifying the *scale* parameter if the HURDLE model is considered (if *scale* = *"median"*, the median library size is used to scale counts instead of 10^6):

```
observed_hurdle <- prepareObserved(
    object = ps_stool_16S,
    scale = "median")
head(observed_hurdle)
```

```
##                            Y      Y0
## OTU_97.192   3.21387169 0.31250
## OTU_97.1666  0.03923904 0.96875
## OTU_97.31213 4.88350981 0.09375
## OTU_97.39094 4.89778959 0.75000
## OTU_97.40451 7.51646781 0.00000
## OTU_97.19587 5.26661203 0.06250
```

Which are different from the non-scaled observed values:

```
head(prepareObserved(object = ps_stool_16S))
```

```
##                            Y      Y0
## OTU_97.192   3.34109346 0.31250
## OTU_97.1666  0.03077166 0.96875
## OTU_97.31213 4.77675725 0.09375
## OTU_97.39094 4.44081133 0.75000
## OTU_97.40451 7.53824550 0.00000
## OTU_97.19587 5.36801832 0.06250
```

The function to compute MD and ZPD values, is `meanDifferences()`:

```
head(meanDifferences(
    estimated = example_HURDLE,
    observed = observed_hurdle
))
```

```
##           MD           ZPD
## 1 -1.0612798  0.0002600107
## 2         NA -0.0010681206
## 3 -0.8258883  0.0007597716
## 4 -3.7745917 -0.0003641001
```

```
## 5 -0.1889343  0.0019494545
## 6 -0.5496976  0.0008419888
```

A wrapper function to simultaneously perform the estimates and the mean differences is `fitModels()`:

```
GOF_stool_16S <- fitModels(
    object = ps_stool_16S,
    models = c("NB", "ZINB", "DM", "ZIG", "HURDLE"),
    scale_HURDLE = c("median", "default"),
    verbose = FALSE # TRUE is always suggested
)
```

Exploiting the internal structure of the `fitModels()`'s output the Root Mean Squared Error (RMSE) values for MD values can be extracted (the lower, the better):

```
plotRMSE(GOF_stool_16S, difference = "MD", plotIt = FALSE)
```

```
##            Model       RMSE
## 1             NB 0.07057518
## 2           ZINB 0.14298816
## 3             DM 0.97766699
## 4            ZIG 0.53150840
## 5  HURDLE_median 0.85437589
## 6 HURDLE_default 3.11439757
```

Similarly, they are extracted for ZPD values:

```
plotRMSE(GOF_stool_16S, difference = "ZPD", plotIt = FALSE)
```

```
##            Model         RMSE
## 1             NB 0.0741340631
## 2           ZINB 0.0219382366
## 3             DM 0.0436436801
## 4            ZIG 0.1129285606
## 5  HURDLE_median 0.0008776059
## 6 HURDLE_default 0.0008832596
```

2.4 Visualization

2.4.1 Mean Differences

To plot estimated and observed values the `plotMD()` function can be used (Figure 2). No systematic trend are expected, moreover, the closer the values to the dotted line are (representing equality between observed and estimated values), the better the goodness of fit relative to the model.

```
plotMD(
    data = GOF_stool_16S,
    difference = "MD",
    split = TRUE
)
```

If some warning messages are shown with this graph, they are likely due to sparse taxa. To address this, the number of NA values generated by each model can be investigated (which are 24 for each HURDLE model):

```
plyr::ldply(GOF_stool_16S, function(model)
    c("Number of NAs" = sum(is.na(model))),
    .id = "Distribution")
```

To summarize the goodness of fit, the Root Mean Squared Error (RMSE) metric is also displayed for each model. For the *HURDLE_default* model, a quite different range of values of mean differences is displayed

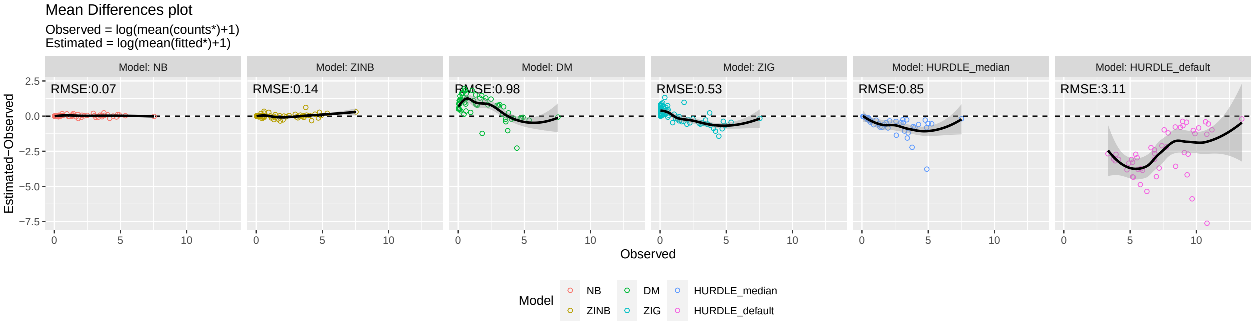

Figure 2: MD plot. Mean-difference (MD) between the estimated and observed count values for each distribution.

because of the excessive *default* scaling proposed (1 million). It is also possible to plot only a subset of the estimated models (Figure 3).

```
plotMD(
    data = GOF_stool_16S[1:5],
    difference = "MD",
    split = TRUE
)
```

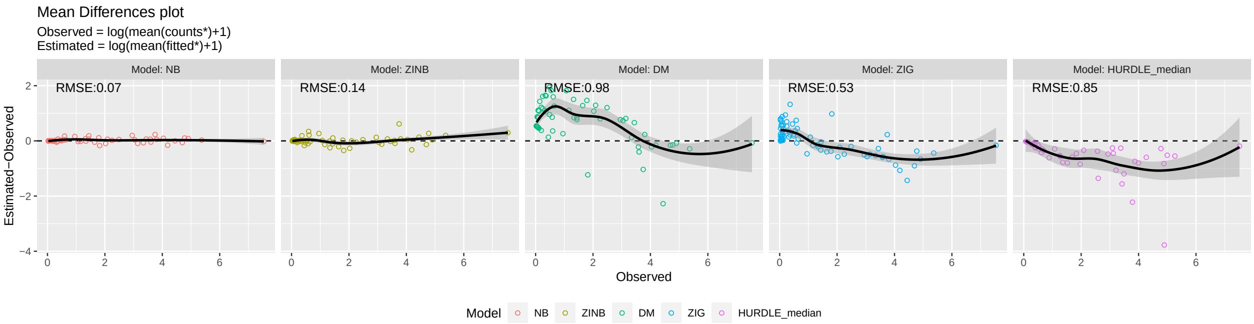

Figure 3: MD plot reduced. Mean-difference (MD) between the estimated and observed count values for the first 5 distributions.

From the Figure 3, *DM* distribution slightly overestimates the logarithm of the average counts for low values, while the *HURDLE_median* distribution presents an overestimation that increases as the observed values increase. *ZIG*, but especially *NB* and *ZINB* distributions produce very similar estimated and observed values. Similarly, to plot the mean differences for Zero Probability/Proportion the `plotMD()` function is used (Figure 4).

```
plotMD(
    data = GOF_stool_16S[1:5],
    difference = "ZPD",
    split = TRUE
)
```

From the figure 4, *ZIG* and *NB* models underestimate the probability to observe a zero for sparse features, while the *HURDLE_median* model presents a perfect fit as the probability to observe a zero is the zero rate itself by construction. *DM* and *ZINB* models produce estimated values very similar to the observed ones. MDs and ZPDs are also available in the Figure 5 with a different output layout.

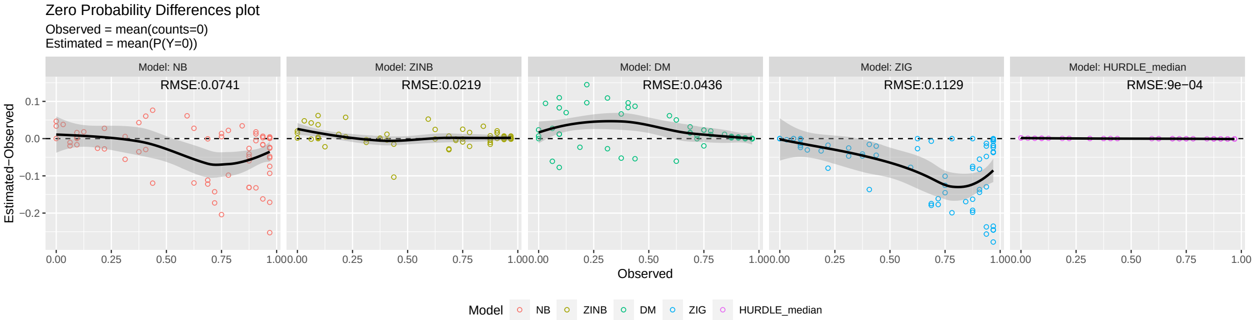

Figure 4: ZPD plot. Mean-difference between the estimated probability to observe a zero and the observed proportion of zero values (ZPD) for the first 5 distributions.

```
plot_grid(plotMD(data = GOF_stool_16S[1:5], difference = "MD", split = FALSE),
    plotMD(data = GOF_stool_16S[1:5], difference = "ZPD", split = FALSE),
    ncol = 2
)
```

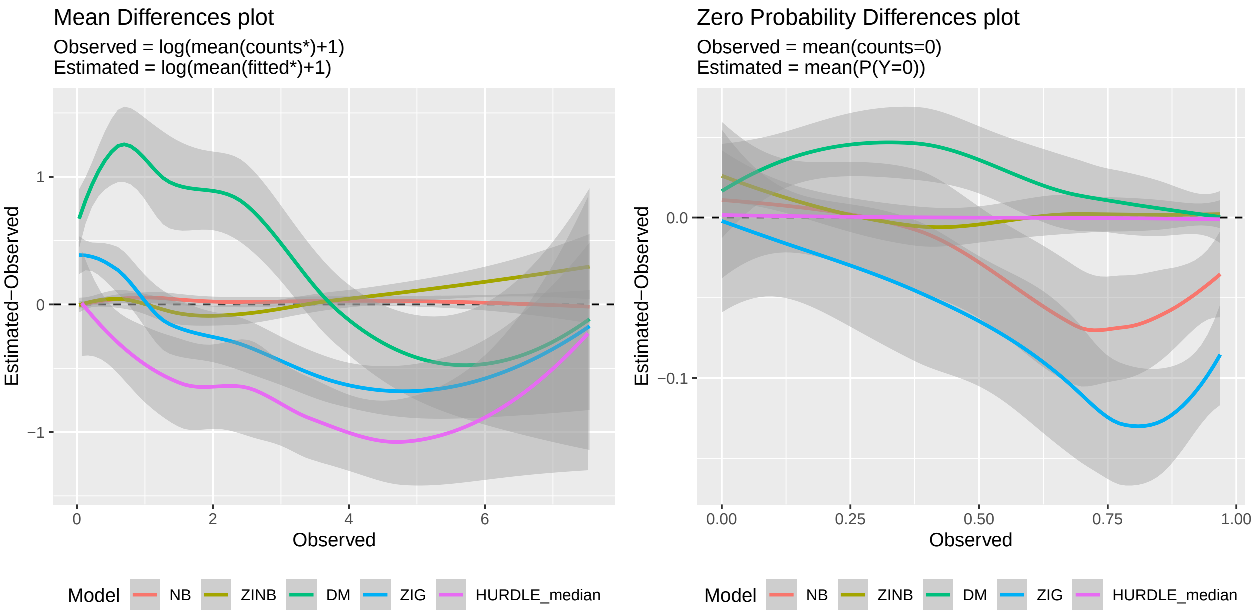

Figure 5: MD and ZPD plots. MD and ZPD plotted together for the first 5 distributions.

2.4.2 RMSE

As already mentioned, to summarize the goodness of fit, the Root Mean Squared Error (RMSE) metric is used. The summary statistics for the overall performance are visible in Figure 6.

```
plot_grid(plotRMSE(GOF_stool_16S, difference = "MD"),
    plotRMSE(GOF_stool_16S, difference = "ZPD"),
    ncol = 2
)
```

The lower the RMSE value, the better the goodness of fit of the model.

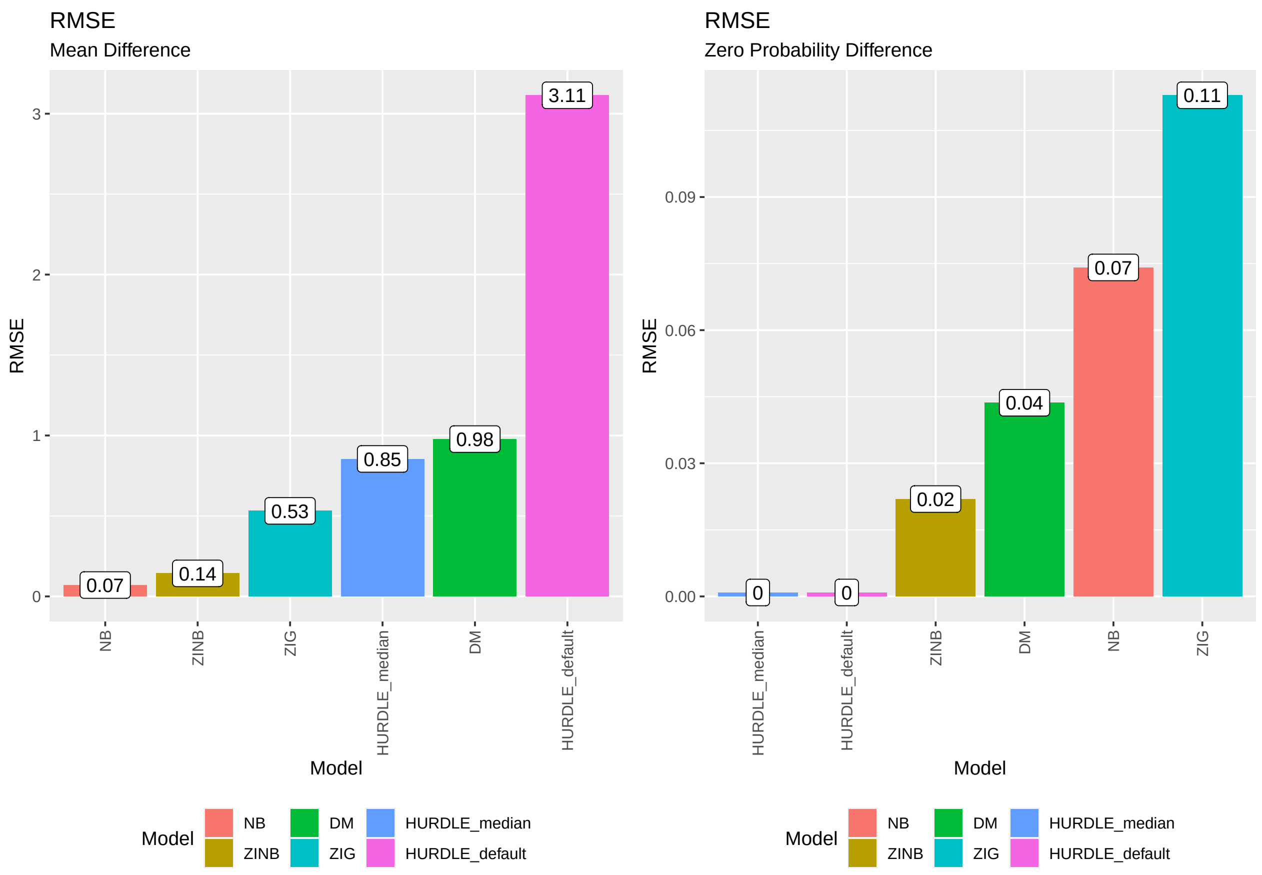

Figure 6: RMSE plot. Root Mean Squared Errors (RMSE) for both the MD and ZPD values for all the distributions.

2.5 Discussion about GOF

The Goodness of Fit chapter is focused on some existing parametric models: NB, ZINB, HURDLE, ZIG, DM. The assumption of this analysis is that if a model estimates the data well, then a method based on that model may be a possibly good choice for studying the differential abundance. Other distributions could also be investigated (Poisson, Zero-Inflated Poisson…) but what about DA methods which are based on non-parametric models such as ANCOM? GOF framework can't be used to compare the parametric models to non-parametric models. However, non-parametric methods may work well in real scenarios due to their added robustness and other evaluations are necessary in order not to favor one group of methods over another.

3 DA methods

Differential abundance analysis is the core of `benchdamic`. DA analysis steps can be performed both directly, using the `DA_<name_of_the_method>()` methods, or indirectly, using the `set_<name_of_the_method>()` functions.

`set_<name_of_the_method>()` functions allow to create lists of instructions for DA methods which can be used by the `runDA()`, `runMocks()`, and `runSplits()` functions (more details in each chapter).

This framework grants a higher flexibility allowing users to set up the instructions for many DA methods only at the beginning of the analysis. If some modifications are needed, the users can re-set the methods or modify the list of instructions directly.

A list of the available methods is presented below (Table 1). They are native to different application fields such as RNA-Seq, single-cell RNA-Seq, or Microbiome data analysis. Some basic information are reported for each DA method, for more details please refer to functions' manual.

Table 1: DA methods available in benchdamic.

**Method (package)**	**Short description**	**Test**	**Normalization / Transformation**	**Suggested input**	**Output**	**Application**
DA_basic (stats)	Simple Student's t and Wilcox tests are used to assess differences between groups	test = 't', 'wilcox'	No normalization	all types	p-values and statistics	General
DA_edgeR (edgeR)	A Negative Binomial (NB) generalized linear model is used to describe the counts	Empirical Bayes + moderated t, robust estimation of priors (if robust = TRUE), with or without weights (generated by weights_ZINB)	norm = 'TMM', 'TMMwsp', 'RLE', 'upperquartile', 'posupperquartile', 'none' (produced by norm_edgeR)	raw counts with edgeR normalization factors	p-values and statistics	RNA-Seq
DA_limma (limma)	A Linear model of the log2-transformed CPMs with weights is used to describe differences between groups	Empirical Bayes + moderated t, with or without weights (generated by weights_ZINB)	norm = 'TMM', 'TMMwsp', 'RLE', 'upperquartile', 'posupperquartile', 'none' (produced by norm_edgeR)	raw counts with edgeR normalization factors	p-values and statistics	RNA-Seq and Microarray
DA_DESeq2 (DESeq2)	A Negative Binomial (NB) generalized linear model is used to describe the counts	Empirical Bayes + LRT, with or without weights (generated by weights_ZINB)	norm = 'ratio', 'poscounts', 'iterate' (produced by norm_DESeq2)	raw counts with DESeq2 size factors	p-values and statistics	RNA-Seq
DA_NOISeq (NOISeq)	A non-parametric approach for the comparison of tag-wise statistics and a noise distribution to detect differential abundance	M and D statistics compared with the noise distribution	norm = 'rpkm', 'uqua', 'tmm', 'n'	raw counts or normal-ized/transformed counts setting norm = "n"	adjusted p-values and statistics	RNA-Seq
DA_dearseq (dearseq)	A variance component score test accounting for data heteroscedasticity through precision weights is used to assess differences between groups	test = 'asymptotic', 'permutation'	automatically transforms raw counts into log(CPM) if preprocessed = FALSE	raw counts or log transformed counts setting preprocessed = TRUE	p-values	RNA-Seq
DA_metagenomeSeq (metagenomeSeq)	Zero-Inflated Gaussian (ZIG) mixture model or Zero-Inflated Log-Gaussian (ZILG) mixture model are used to describe the counts	model = 'fitZig', 'fitFeatureModel'	norm = 'CSS' (produced by norm_CSS)	raw counts with CSS normalization factors	p-values and statistics	Microbiome
DA_corncob (corncob)	A Beta-Binomial regression model is used to descrive the relative counts	test = 'LRT', 'Wald' with (if boot = TRUE) or without bootstrap	Automatically transforms raw counts into relative abundances (like using TSS)	raw counts	p-values and statistics	Microbiome
DA_ALDEx2 (ALDEx2)	Compositional approach - Monte-Carlo sampling from a Dirichlet distribution to estimate the real relative abundances and CLR-like transformation to assess differences between groups	test = 't', 'wilcox', 'kw', 'ANOVA', 'glm'	denom = 'all', 'iqlr', 'zero', 'lvha', 'median', or decided by the user. With test = 'glm', denom = 'all'	raw counts	p-values and statistics	Microbiome
DA_ANCOM (ANCOMBC)	Compositional approach - Analysis of microbiome compositions with or without "sampling fraction" bias correction	ANCOM-II ANOVA models for log-ratios (if BC = FALSE) or linear model with offsets for log-counts (if BC = TRUE)	Automatic in-method transformation and zero types imputation	raw counts	p-values and statistics if BC = TRUE, only statistics otherwise	Microbiome
DA_Seurat (Seurat)	A test is performed on transformed, normalized, and/or scaled data to assess differences between groups	test = 'wilcox', 'bimod', 'roc', 't', 'negbinom', 'poisson', 'LR', 'MAST', 'DESeq2'	norm = 'LogNormalize', 'CLR', 'RC', 'none' (with test = 'negbinom', 'poisson', or 'DESeq2', data are not transformed). scale.factor parameter to scale data	raw counts	p-values and statistics	scRNA-Seq
DA_MAST (MAST)	A Truncated Gaussian hurdle model is used to describe the counts	Standard Empirical Bayes + LRT	log(CPM) transformation + in-method normalization	raw counts	p-values and statistics	scRNA-Seq

Please remember that the data pre-processing, including QC analysis, filtering steps, and normalization, are not topics treated in `benchdamic`. In real life situations those steps precede the DA analysis and they are of extreme importance to obtain reliable results.

Some exceptions are present for the normalization step. In `benchdamic`, `norm_edgeR()`, `norm_DESeq2()`, `norm_CSS()`, and `norm_TSS()` are implemented functions to add the normalization/scaling factors to the `phyloseq` or `TreeSummarizedExperiment` objects, needed by DA methods. As for DA methods, normalization instructions list, including the previous functions, can be set using `set_<normalization_name>()` or `setNormalizations()` too. To run the normalization instructions the function `runNormalizations()` can be used (more examples will follow).

Many DA methods already contain options to normalize or transform counts. If more complex normalizations/transformations are needed, all the DA methods support the use of `TreeSummarizedExperiment` objects. In practice, users can put the modified count matrix in a named assay (the *counts* assay is the default one which contains the raw counts) and run the DA method on that assay using the parameter *assay_name = "assay_to_use"*.

3.1 Add a custom DA method

To add a custom method to the benchmark, it must:

- include a *verbose = TRUE* (or *FALSE*) parameter to let the user know what the method is doing;
- return a *pValMat* matrix which contains the raw p-values and adjusted p-values in *rawP* and *adjP* columns respectively;
- return a *statInfo* matrix which contains the summary statistics for each feature, such as the *logFC*, standard errors, test statistics and so on;
- return a *name* which contains the complete name of the used method.

An example is proposed:

```
DA_yourMethod <- function(
        object,
        assay_name = "counts",
        param1,
        param2,
        verbose = TRUE)
{
    if(verbose)
        message("Reading data")
    # Extract the data from phyloseq or TreeSummarizedExperiment
    counts_metadata <- get_counts_metadata(
        object = object,
        assay_name = assay_name)
    counts <- counts_metadata[[1]] # First position = counts
    metadata <- counts_metadata[[2]] # Second position = metadata

    ### your method's code
    # Many things here
    if(verbose)
        message("I'm doing this step.")
    # Many other things here
    ### end of your method's code

    if(verbose)
        message("Extracting important statistics")
```

```r
    vector_of_pval <- NA # contains the p-values
    vector_of_adjusted_pval <- NA # contains the adjusted p-values
    name_of_your_features <- NA # contains the OTU, or ASV, or other feature
                                 # names. Usually extracted from the rownames of
                                 # the count data
    vector_of_logFC <- NA # contains the logFCs
    vector_of_statistics <- NA # contains other statistics

    if(verbose)
        message("Preparing the output")
    pValMat <- data.frame("rawP" = vector_of_pval,
                          "adjP" = vector_of_adjusted_pval)
    statInfo <- data.frame("logFC" = vector_of_logFC,
                           "statistics" = vector_of_statistics)
    name <- "write.here.the.name"
    # Be sure that the algorithm hasn't changed the order of the features. If it
    # happens, re-establish the original order.
    rownames(pValMat) <- rownames(statInfo) <- name_of_your_features

    # Return the output as a list
    return(list("pValMat" = pValMat, "statInfo" = statInfo, "name" = name))
} # END - function: DA_yourMethod
```

Once the custom method is set, it can be run by using the `DA_yourMethod()` function or manually, by setting a list of instructions of the custom method with the desired combination of parameters:

```r
my_custom_method <- list(
    customMethod.1 = list( # First instance
        method = "DA_yourMethod", # The name of the function to call
        assay_name = "counts",
        param1 = "A", # Its combination of parameters
        param2 = "B"), # No need of verbose and object parameters
    customMethod.2 = list( # Second instance
        method = "DA_yourMethod",
        assay_name = "counts",
        param1 = "C",
        param2 = "D")
    # Other istances
)
```

The *method* field, containing the name of the method to call is mandatory, while the *verbose* parameter is not necessary. Instead, the *object* is not needed in order to keep the `my_custom_method` object only a list of instructions.

4 Type I Error Control

Assumption: Many DA methods do not control the number of false discoveries.

Research question: Which are the DA methods which can control the number of false positives in your data?

4.1 TIEC structure

The Type I Error is the probability of a statistical test to call a feature DA when it is not, under the null hypothesis. To evaluate the Type I Error rate Control (TIEC) for each differential abundance detection

method:

1. using the `createMocks()` function, homogeneous samples (*e.g.*, only the samples from one experimental group) are randomly assigned to a group ('grp1' or 'grp2');
2. DA methods are run to find differences between the two mock groups using `runMocks()`;
3. the number of DA feature for each method is counted, these are False Positives (FP) by construction;
4. points 1-3 are repeated many times ($N = 10$, but at least 1000 is suggested) and the results are averaged using the `createTIEC()` function.

In this setting, the p-values of a perfect test should be uniformly distributed between 0 and 1 and the false positive rate (FPR or observed α), which is the observed proportion of significant tests, should match the nominal value (*e.g.*, $\alpha = 0.05$).

The relationships between the functions used in this section are explained by the diagram in Figure 7.

4.2 Create mock comparisons

Using `createMocks()` function, samples are randomly grouped, `N = 10` times. A higher `N` is suggested (at least 1000) but in that case a longer running time is required.

```
set.seed(123)
my_mocks <- createMocks(
    nsamples = phyloseq::nsamples(ps_stool_16S),
    N = 10
) # At least N = 1000 is suggested
```

4.3 Set up normalizations and DA methods

Once the mocks have been generated, DA analysis is performed. Firstly, some normalization factors, such as *TMM* from `edgeR` and *CSS* from `metagenomeSeq`, and some size factors such as *poscounts* from `DESeq2` are added to the `phyloseq` object (or `TreeSummarizedExperiment` object). This can be done, manually, using the `norm_edgeR()`, `norm_DESeq2()`, and `norm_CSS()` methods:

```
ps_stool_16S <- norm_edgeR(
    object = ps_stool_16S,
    method = "TMM"
)
ps_stool_16S <- norm_DESeq2(
    object = ps_stool_16S,
    method = "poscounts"
)
ps_stool_16S <- norm_CSS(
    object = ps_stool_16S,
    method = "CSS"
)
```

Or automatically, using the `setNormalizations()` and `runNormalizations()` methods:

```
my_normalizations <- setNormalizations(
    fun = c("norm_edgeR", "norm_DESeq2", "norm_CSS"),
    method = c("TMM", "poscounts", "CSS"))
ps_stool_16S <- runNormalizations(normalization_list = my_normalizations,
    object = ps_stool_16S, verbose = TRUE)
```

Some messages "Found more than one"phylo" class in cache…" could be shown after running the previous functions. They are caused by duplicated class names between `phyloseq` and `tidytree` packages and can be

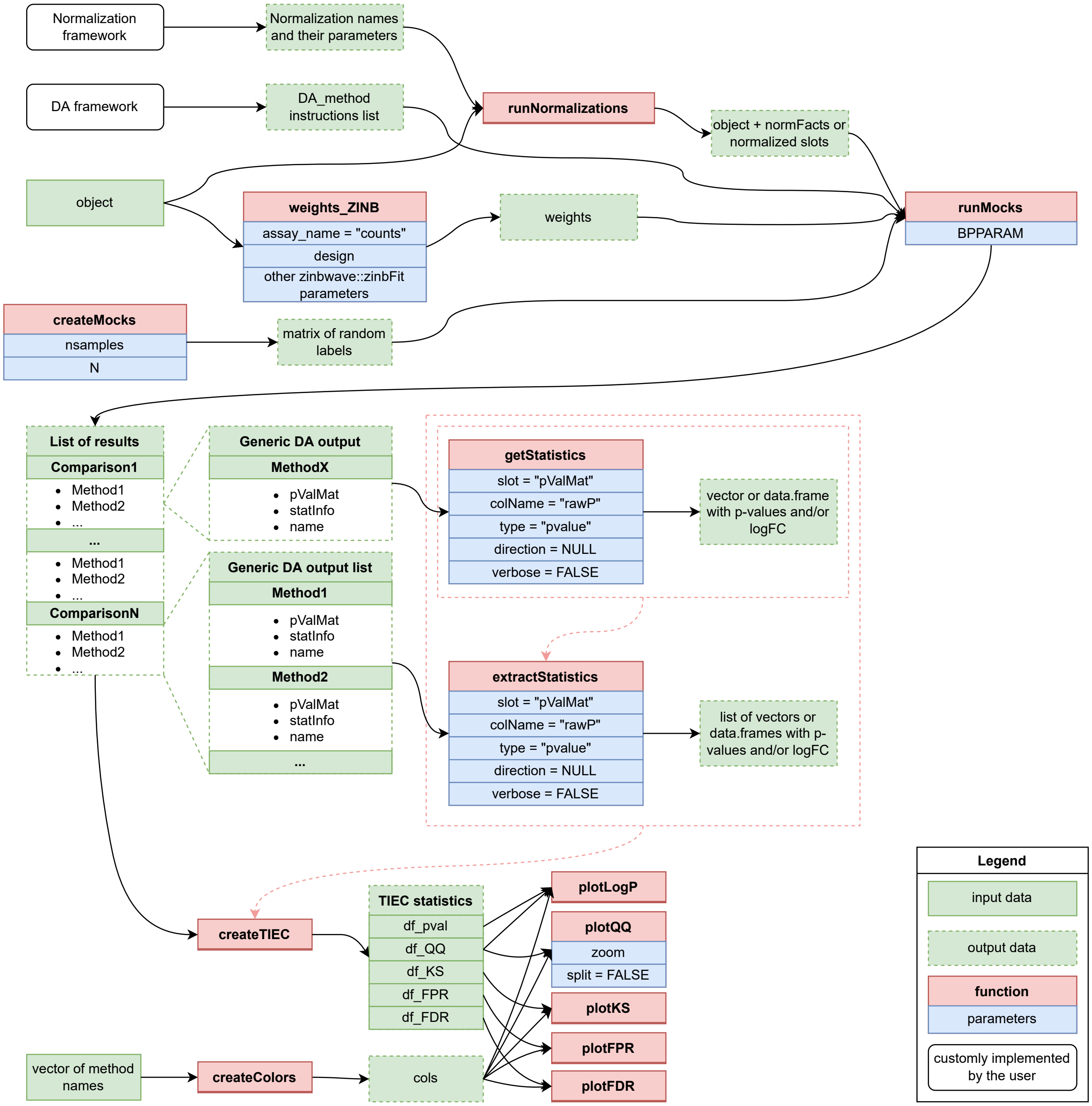

Figure 7: Type I Error Control diagram.

ignored.

After the normalization/size factors have been added to the phyloseq or TreeSummarizedExperiment object, the user could decide to filter rare taxa which do not carry much information. In this example vignette a simple filter is applied to keep only features with a count in at least 3 samples:

```
ps_stool_16S <- phyloseq::filter_taxa(
    physeq = ps_stool_16S,
    flist = function(x) sum(x > 0) >= 3, prune = TRUE)
ps_stool_16S
```

```
## phyloseq-class experiment-level object
## otu_table()   OTU Table:          [ 47 taxa and 32 samples ]
## sample_data() Sample Data:        [ 32 samples by 10 sample variables ]
## tax_table()   Taxonomy Table:     [ 47 taxa by 6 taxonomic ranks ]
## phy_tree()    Phylogenetic Tree: [ 47 tips and 46 internal nodes ]
```

Some zero-inflated negative binomial weights using the weights_ZINB() function are computed. They can be used as observational weights in the generalized linear model frameworks of DA_edgeR(), DA_DESeq2(), and DA_limma(), as described in (Van den Berge *et al.*, 2018).

```
zinbweights <- weights_ZINB(
    object = ps_stool_16S,
    K = 0,
    design = "~ 1",
)
```

For each row of the mock_df data frame a bunch of DA methods is run. In this demonstrative example the following DA methods are used:

- basic *t* and *wilcox* tests;
- edgeR with *TMM* scaling factors (Robinson *et al.*, 2010) with and without *ZINB* weights (Risso *et al.*, 2018; Van den Berge *et al.*, 2018);
- DESeq2 with *poscounts* normalization factors (Love *et al.*, 2014) with and without *ZINB* weights (Risso *et al.*, 2018; Van den Berge *et al.*, 2018);
- limma-voom with *TMM* scaling factors (Ritchie *et al.*, 2015; Law *et al.*, 2014; Phipson *et al.*, 2016) with and without *ZINB* weights (Risso *et al.*, 2018; Van den Berge *et al.*, 2018);
- ALDEx2 with *all* and *iqlr* data transformation (*denom* parameter) performing the *wilcox* test (Fernandes *et al.*, 2014);
- metagenomeSeq with *CSS* normalization factors using both the *fitFeatureModel* (for a zero-inflated log-normal distribution, mixture model, as suggested in the package vignette) and the *fitZig* (for a zero-inflated gaussian distribution, mixture model) algorithms (Paulson *et al.*, 2013);
- corncob with a focus on average differences (not dispersion, regulated by *phi.formula* and *phi.formula_null* parameters) using both *Wald* and *LRT* tests (Martin *et al.*, 2020);
- MAST with both rescalings, *default* (*i.e.* 10^6, for CPMs) and *median* (Finak *et al.*, 2015);
- Seurat with *LogNormalize* and *CLR* normalization/transformations, *t* and *wilcox* tests, and 10^5 as scaling factor (Butler *et al.*, 2018);
- ANCOM based on ANCOM-II algorithm with sampling fraction bias correction (*BC* parameter) (Lin and Peddada, 2020; Kaul *et al.*, 2017);
- dearseq with *permutation* and *asymptotic* tests (Gauthier *et al.*, 2020);

Among the available methods, `NOISeq` (Tarazona *et al.*, 2015) has not been used since it does not return p-values but only adjusted ones. Many combination of parameters are still possible for all the methods.

```
my_basic <- set_basic(pseudo_count = FALSE,
    contrast = c("group", "grp2", "grp1"),
    test = c("t", "wilcox"),
    paired = FALSE,
    expand = TRUE)

my_edgeR <- set_edgeR(
    pseudo_count = FALSE,
    group_name = "group",
    design = ~ group,
    robust = FALSE,
    coef = 2,
    norm = "TMM",
    weights_logical = c(TRUE, FALSE),
    expand = TRUE)

my_DESeq2 <- set_DESeq2(
    pseudo_count = FALSE,
    design = ~ group,
    contrast = c("group", "grp2", "grp1"),
    norm = "poscounts",
    weights_logical = c(TRUE, FALSE),
    alpha = 0.05,
    expand = TRUE)

my_limma <- set_limma(
    pseudo_count = FALSE,
    design = ~ group,
    coef = 2,
    norm = "TMM",
    weights_logical = c(FALSE, TRUE),
    expand = TRUE)

my_ALDEx2 <- set_ALDEx2(
    pseudo_count = FALSE,
    design = "group",
    mc.samples = 128,
    test = "wilcox",
    paired.test = FALSE,
    denom = c("all", "iqlr"),
    contrast = c("group", "grp2", "grp1"),
    expand = TRUE)

my_metagenomeSeq <- set_metagenomeSeq(
    pseudo_count = FALSE,
    design = "~ group",
    coef = "groupgrp2",
    norm = "CSS",
    model = c("fitFeatureModel", "fitZig"),
    expand = TRUE)
```

```
my_corncob <- set_corncob(
    pseudo_count = FALSE,
    formula = ~ group,
    formula_null = ~ 1,
    phi.formula = ~ group,
    phi.formula_null = ~ group,
    test = c("Wald", "LRT"),
    boot = FALSE,
    coefficient = "groupgrp2")

my_MAST <- set_MAST(
    pseudo_count = FALSE,
    rescale = c("default", "median"),
    design = "~ 1 + group",
    coefficient = "groupgrp2",
    expand = TRUE)

my_Seurat <- set_Seurat(
    pseudo_count = FALSE,
    test = c("t", "wilcox"),
    contrast = c("group", "grp2", "grp1"),
    norm = c("LogNormalize", "CLR"),
    scale.factor = 10^5,
    expand = TRUE
)

my_ANCOM <- set_ANCOM(
    pseudo_count = FALSE,
    fix_formula = "group",
    contrast = c("group", "grp2", "grp1"),
    BC = TRUE,
    expand = TRUE
)

my_dearseq <- set_dearseq(
    pseudo_count = FALSE,covariates = NULL,
    variables2test = "group",
    preprocessed = FALSE,
    test = c("permutation", "asymptotic"),
    expand = TRUE)

my_methods <- c(my_basic, my_edgeR, my_DESeq2, my_limma, my_metagenomeSeq,
    my_corncob, my_ALDEx2, my_MAST, my_Seurat, my_ANCOM, my_dearseq)
```

After concatenating all the DA instructions, they are run on the mock comparisons using the `runMocks()` function:

```
# Random grouping each time
Stool_16S_mockDA <- runMocks(
    mocks = my_mocks,
    method_list = my_methods,
    object = ps_stool_16S,
    weights = zinbweights,
    verbose = FALSE)
```

If some warnings are reported, *verbose = TRUE* can be used to obtain the method name and the mock comparison where the warnings occured.

The structure of the output in this example is the following:

- *Comparison1* to *Comparison10* on the first level, which contains:
  - *Method1* to *Method23* output lists on the second level:
    * *pValMat* which contains the matrix of raw p-values and adjusted p-values in *rawP* and *adjP* columns respectively;
    * *statInfo* which contains the matrix of summary statistics for each feature, such as the *logFC*, standard errors, test statistics and so on;
    * *dispEsts* which contains the dispersion estimates for methods like `edgeR` and `DESeq2`;
    * *name* which contains the complete name of the used method.

The list of methods can be run in parallel leveraging the `BiocParallel` package. In details, parallelization is supported in Linux/Mac OS through the `MulticoreParam()` function (using the FORK implementation), as long as `ANCOM` functions are not included in the list of methods due to a different parallelization management of those functions.

```
bpparam = BiocParallel::MulticoreParam()
Stool_16S_mockDA <- runMocks(
    mocks = my_mocks,
    method_list = my_methods[-21], # Remove ANCOM
    object = ps_stool_16S,
    weights = zinbweights,
    verbose = FALSE,
    BPPARAM = bpparam)
```

`ANCOM` based methods are usually the most time consuming. Parallel computing is still possible as long as it is directly managed by those methods (`n_cl` parameter). In the following example, each mock dataset is analyzed in serial mode but `ANCOM` is run in more than one core.

```
# Modify the n_cl parameter
my_ANCOM_parallel <- set_ANCOM(
    pseudo_count = FALSE,
    fix_formula = "group",
    contrast = c("group", "grp2", "grp1"),
    BC = TRUE,
    n_cl = 2, # Set this number according to your machine
    expand = TRUE
)

bpparam = BiocParallel::SerialParam()
Stool_16S_mockDA_ANCOM <- runMocks(
    mocks = my_mocks,
    method_list = my_ANCOM_parallel, # Only ANCOM
    object = ps_stool_16S,
    weights = zinbweights,
    verbose = FALSE,
    BPPARAM = bpparam)
```

4.3.1 Add a new DA method later in the analysis

It may happen that at a later time the user wants to add to the results already obtained, the results of another group of methods. For example a new version of `limma`:

```
my_new_limma <- set_limma(
    pseudo_count = FALSE,
    design = ~ group,
    coef = 2,
    norm = "CSS",
    weights_logical = FALSE)
```

Which returns a new set of `limma` instructions and a warning for using CSS normalization factors instead of those native to `edgeR`.

First of all, the same mocks and the same object must be used to obtain the new results. To run the new instructions the `runMocks()` function is used:

```
Stool_16S_mockDA_new_limma <- runMocks(
    mocks = my_mocks,
    method_list = my_new_limma,
    object = ps_stool_16S,
    verbose = FALSE)
```

To put everything together a `mapply()` function is used to exploit the output structures:

```
Stool_16S_mockDA_merged <- mapply(
    Stool_16S_mockDA, # List of old results
    Stool_16S_mockDA_new_limma, # List of new results
    FUN = function(old, new){
        c(old, new) # Concatenate the elements
    }, SIMPLIFY = FALSE)
```

4.4 Counting the False Positives

The `createTIEC()` function counts the FPs and evaluates the p-values distributions:

```
TIEC_summary <- createTIEC(Stool_16S_mockDA)
```

A list of 5 `data.frames` is produced:

1. `df_pval` is a 5 columns and *number_of_features* x *methods* x *comparisons* rows data frame. The five columns are called *Comparison*, *Method*, *variable* (which contains the feature names), *pval* and *padj*;
2. `df_FPR` is a 5 columns and *methods* x *comparisons* rows data frame. For each set of method and comparison, the proportion of FPs, considering 3 threshold (0.01, 0.05, 0.1) is reported;
3. `df_FDR` is a 4 columns and number of *methods* rows data frame. For each method, the average False Discovery Rate is computed averaging the results across all comparisons (considering 3 threshold, 0.01, 0.05, and 0.1);
4. `df_QQ` contains the average p-value for each theoretical quantile, *i.e.* the QQ-plot coordinates to compare the mean observed p-values distribution across comparisons, with the theoretical uniform distribution. Indeed, the observed p-values should follow a uniform distribution under the null hypothesis of no differential abundant features presence;
5. `df_KS` is a 5 columns and *methods* x *comparisons* rows data frame. For each set of method and comparison, the Kolmogorov-Smirnov test statistics and p-values are reported in *KS* and *KS_pval* columns respectively.

4.5 Visualization

4.5.1 False Positive Rate

The false positive rate (FPR or observed α), which is the observed proportion of significant tests, should match the nominal value because all the findings are false positive by construction. In this example `edgeR.TMM`, `edgeR.TMM.weighted`, `limma.TMM.weighted`, and `metagenomeSeq.CSS.fitZig` appear to be quite over all the thresholds (liberal behavior), differently `ALDEx2.all.wilcox.unpaired` and `basic_t` methods are below (conservative behavior) or in line with the thresholds (Figure 8).

```
cols <- createColors(variable = levels(TIEC_summary$df_pval$Method))
plotFPR(df_FPR = TIEC_summary$df_FPR, cols = cols)
```

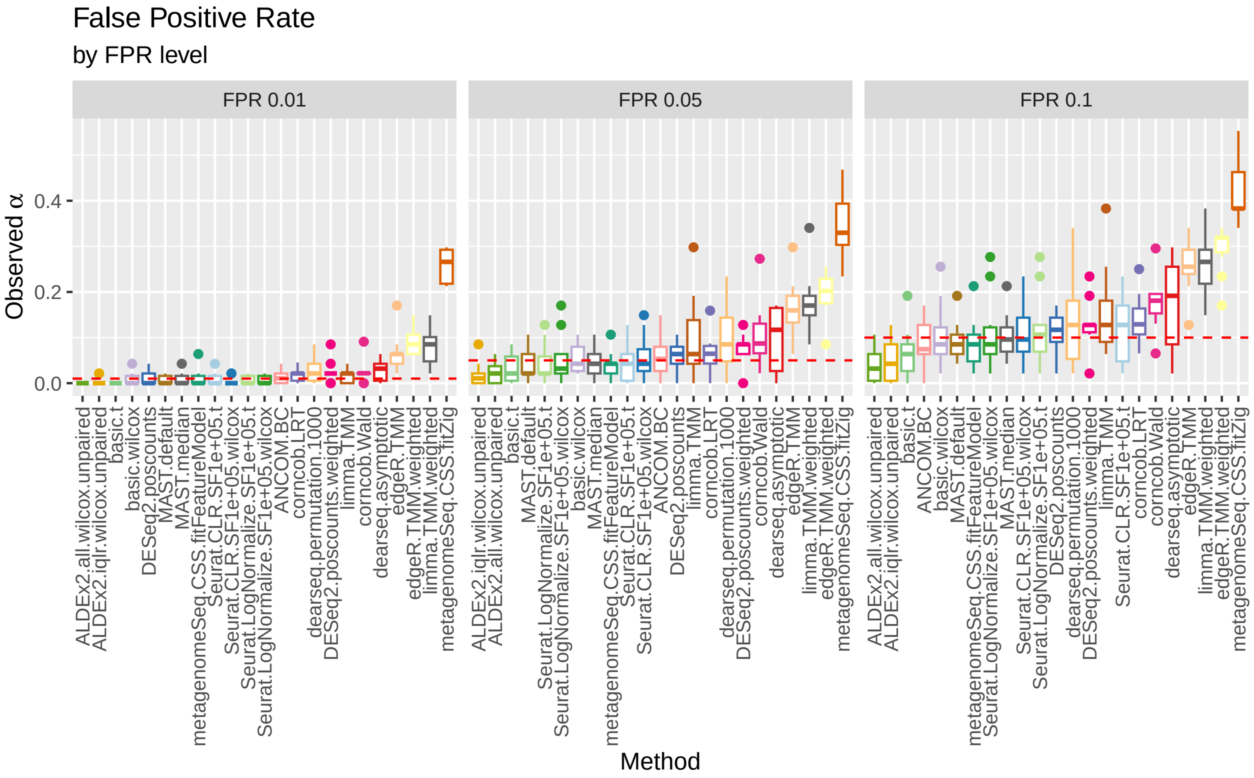

Figure 8: FPR plot. Boxplots of the proportion of raw p-values lower than the commonly used thresholds for the nominal α (i.e. the False Positive Rate) for each DA method.

4.5.2 False Discovery Rate

The false discovery rate $FDR = E\left[\frac{FP}{FP+TP}\right]$ is the expected value of the ratio between the false positives and all the positives. By construction, mock comparisons should not contain any TPs and when all the hypotheses are null, FDR and FWER (Family Wise Error Rate) coincide. For each set of method and comparison, the FDR is set equal to 1 (if at least 1 DA feature is found) or 0 (if no DA features are found). Hence, the estimated FDR is computed by averaging the values across all the mock comparisons. As the number of mock comparisons increases, the more precise the estimated FDR will be. Just as alpha is set as a threshold for the p-value to control the FPR, a threshold for the adjusted p-value, which is the FDR analog of the p-value, can be set. FDR values should match the nominal values represented by the red dashed lines. In this example, the number of mock comparisons is set to 10, so the estimates are unprecise. That said, ALDEx2 based methods, `basic_t`, and `Seurat.CLR.SF1e+05.t` methods are able to control the FDR for all the considered thresholds (Figure 9).

```
plotFDR(df_FDR = TIEC_summary$df_FDR, cols = cols)
```

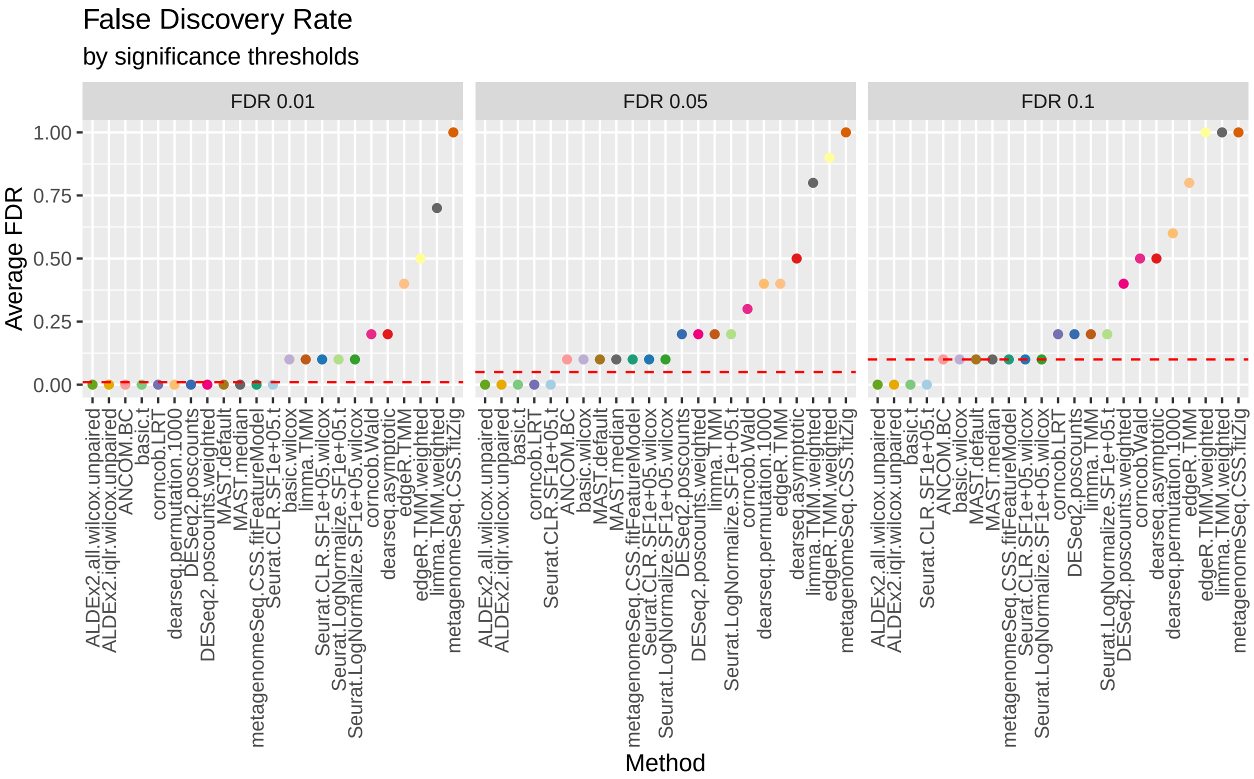

Figure 9: FDR plot. Average nominal False Discovery Rate values for several commonly used thresholds for each DA method.

4.5.3 QQ-Plot

The p-values distribution under the null hypothesis should be uniform. This is qualitatively summarized in the QQ-plot in Figure 10 where the bisector represents a perfect correspondence between observed and theoretical quantiles of p-values. For each theoretical quantile, the corresponding observed quantile is obtained averaging the observed p-values' quantiles from all 10 mock datasets. The plotting area is zoomed-in to show clearly the area between 0 and 0.1.

Methods over the bisector show a conservative behavior, while methods below the bisector a liberal one.

The starting point is determined by the total number of features. In our example the starting point for the theoretical p-values is computed as 1 divided by the number of taxa, rounded to the second digit. In real experiments, where the number of taxa is higher, the starting point is closer to zero.

```
plotQQ(df_QQ = TIEC_summary$df_QQ, zoom = c(0, 0.1), cols = cols) +
    guides(colour = guide_legend(ncol = 1))
```

As the number of methods increases, distinguishing their curves becomes more difficult. For this reason it is also possible to plot each method singularly (Figure 11).

```
plotQQ(df_QQ = TIEC_summary$df_QQ, zoom = c(0, 1), cols = cols, split = TRUE)
```

4.5.4 Kolmogorov-Smirnov test

Departure from uniformity is quantitatively evaluated through the Kolmogorov-Smirnov test which is reported for each method across all mock datasets using the the `plotKS` function in Figure 12.

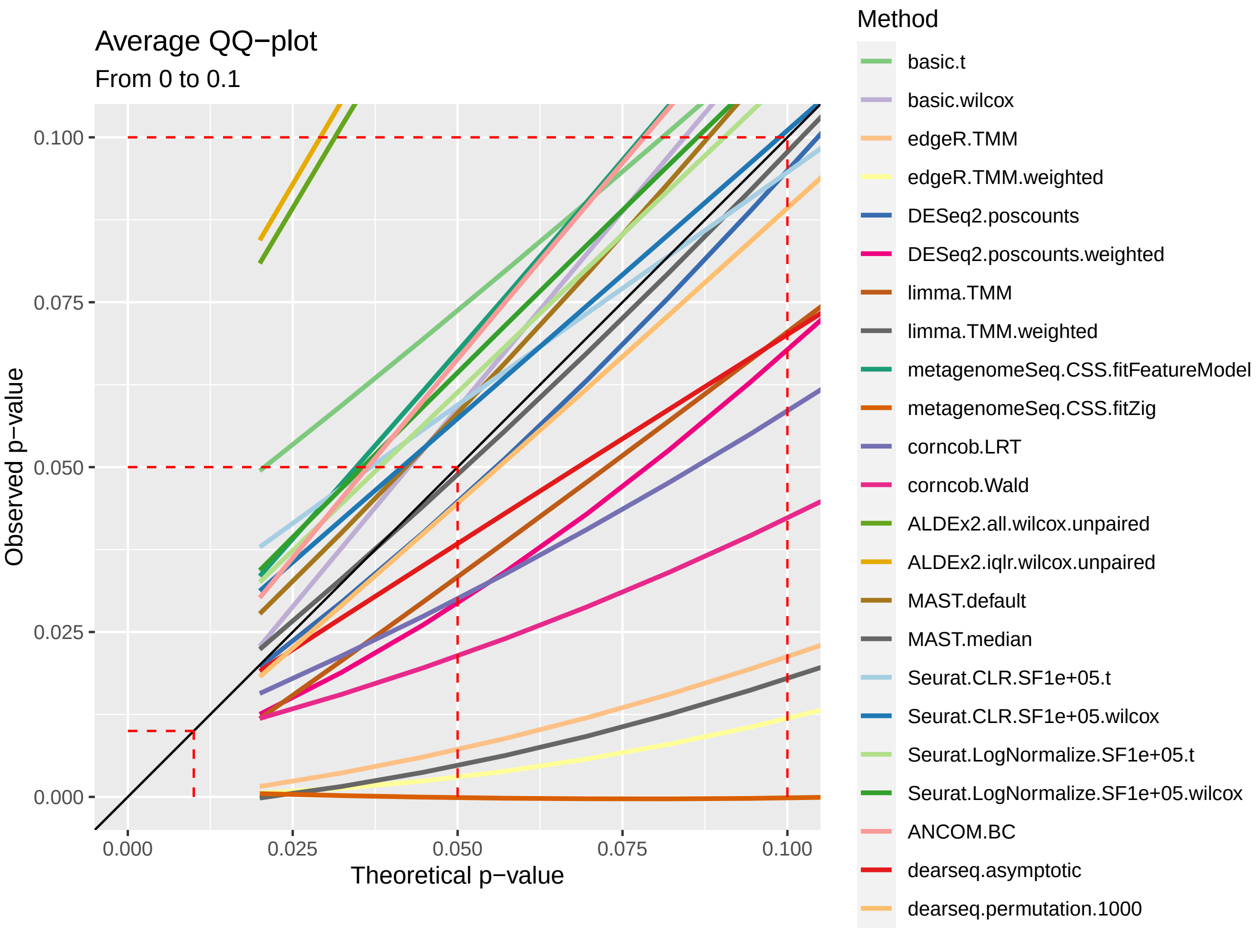

Figure 10: QQ plot. Quantile-quantile plot from 0 to 0.1 for each DA methods. Average curves are reported

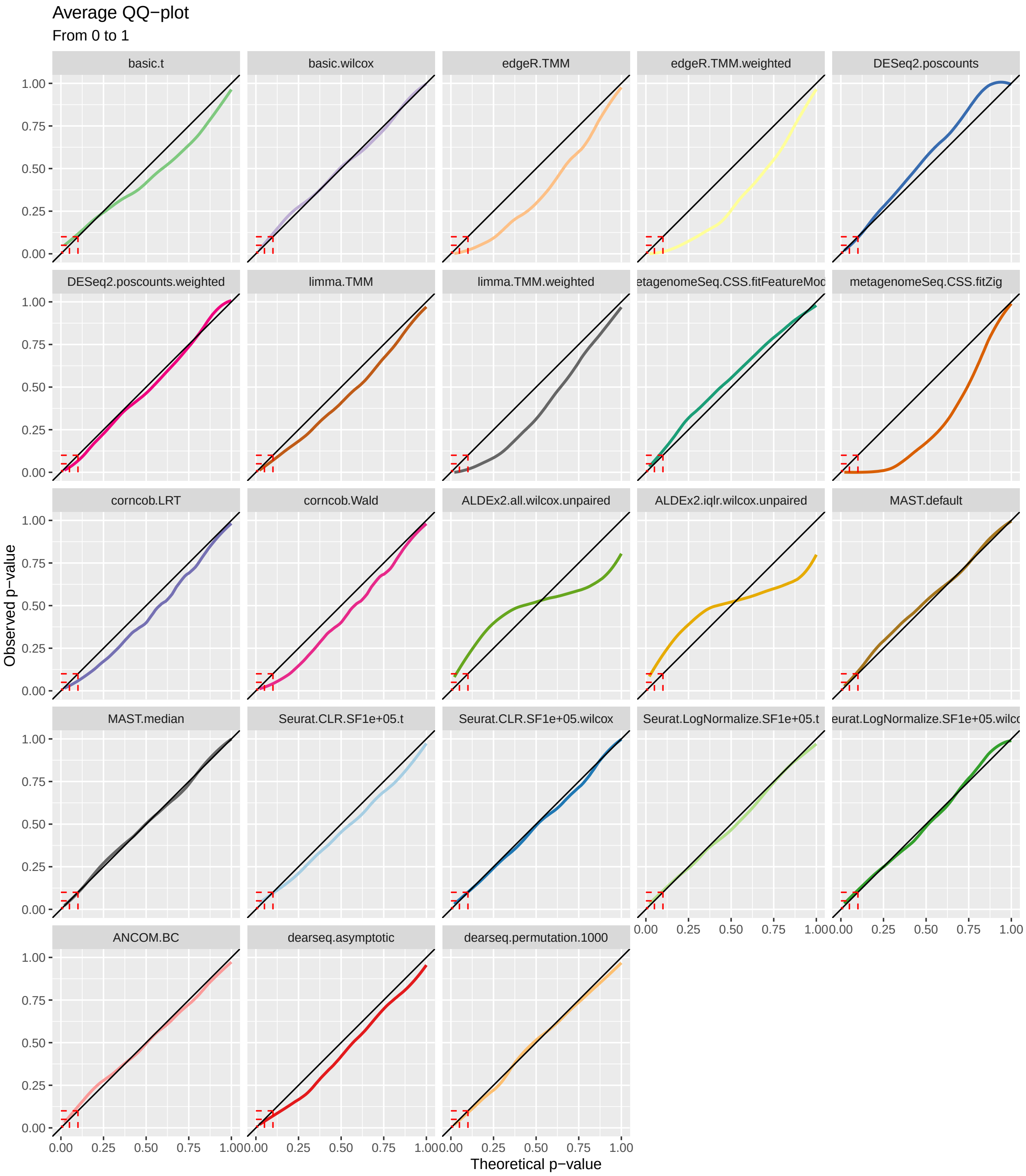

Figure 11: QQ plot. Quantile-quantile plots from 0 to 1 for each DA method are displayed separately. Average curves are reported

```
plotKS(df_KS = TIEC_summary$df_KS, cols = cols)
```

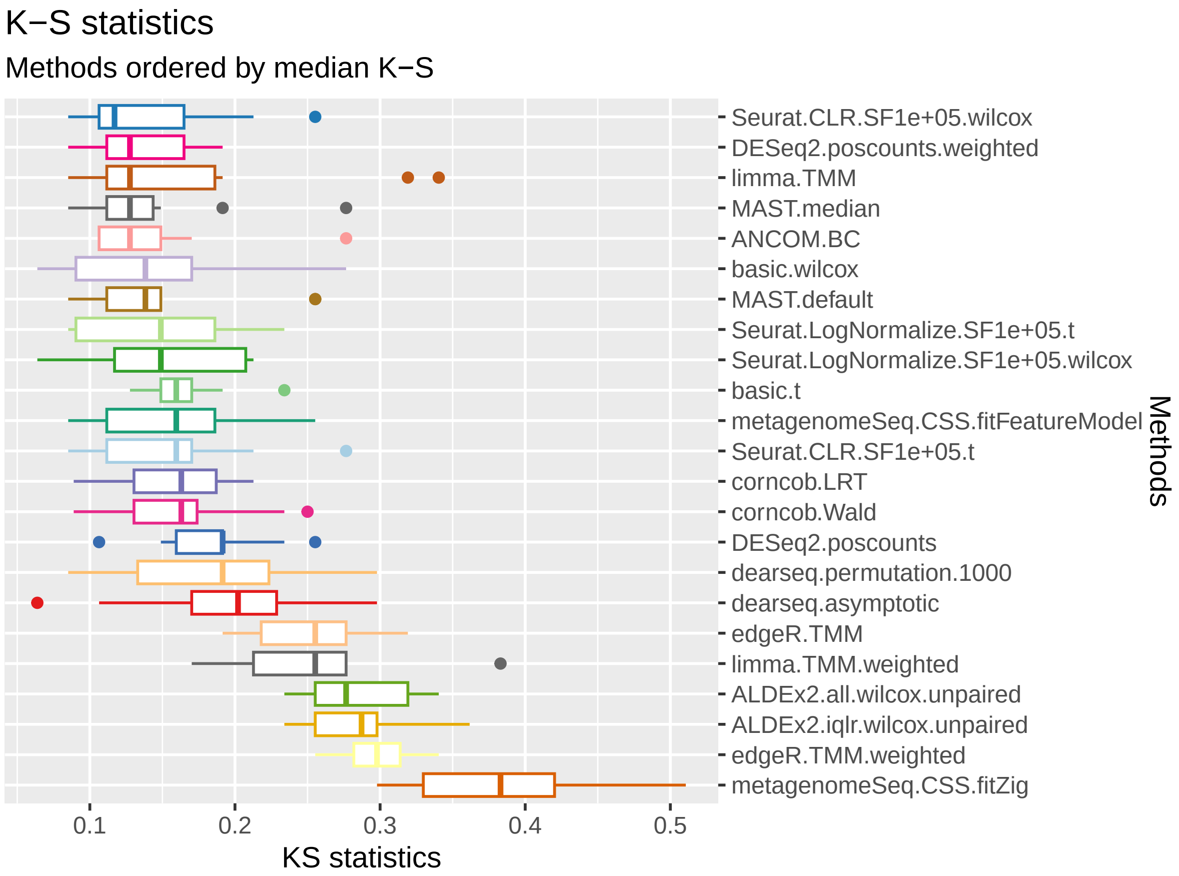

Figure 12: KS plot. Kolmogorov-Smirnov (KS) statistic boxplots for each DA methods where the raw p-values distribution is compared with a uniform distribution.

High KS values indicates departure from the uniformity while low values indicates closeness. All the clues obtained in the previous figures 10 and 11 are confirmed by the KS statistics: `metagenomeSeq.CSS.fitZig`, which was very liberal and its distribution of p-values is the farthest from uniformity among the tested methods. Also ALDEx2 based methods show high KS values, indeed they showed a very conservative behaviour.

4.5.5 Log distribution of p-values

Looking at the p-values' log-scale can also be informative. This is because behavior in the tail may be poor even when the overall p-value distribution is uniform, with a few unusually small p-values in an otherwise uniform distribution. Figure 13 displays the distributions of all the p-values (in negative log scale) generated by each DA method across all the mock comparisons.

```
plotLogP(df_pval = TIEC_summary$df_pval, cols = cols)
```

Similarly, figure 14 exploits the structure of the `df_QQ` data.frame generated by the `createTIEC()` function to display the distribution of the p-values (in negative log scale) generated by each DA method, averaged among mock comparisons (only ten in this example). As this second graphical representation is only based on 1 averaged p-value for each quantile, it is also less influenced by anomalously large values.

```
plotLogP(df_QQ = TIEC_summary$df_QQ, cols = cols)
```

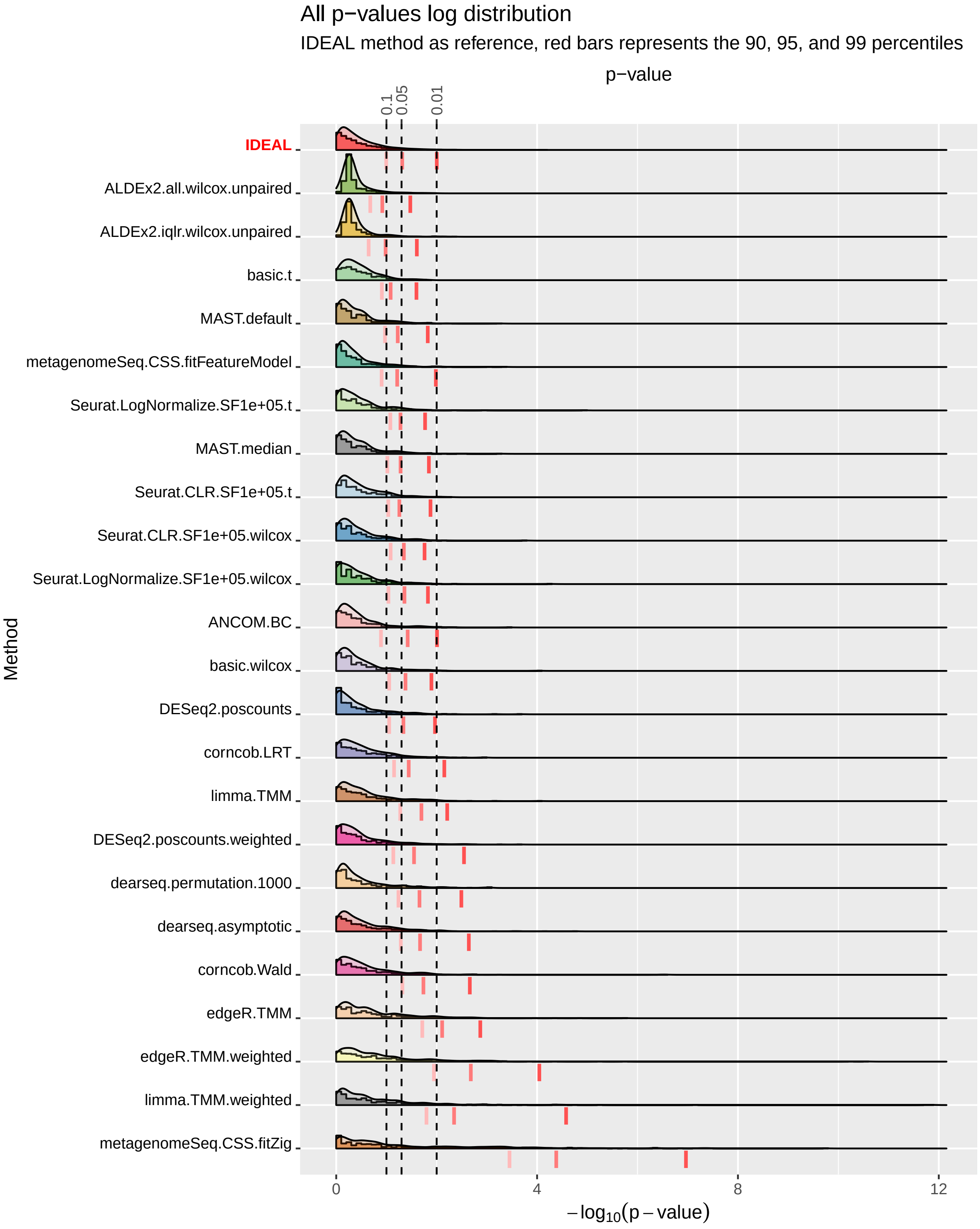

Figure 13: -log10(p-value) plot. Negative logarithm distribution of p-values. Red-shaded vertical bars represent the 90, 95, and 99 percentiles for the negative log distribution of p-values for each method. They should align with the dotted lines which represent the percentiles of the IDEAL distribution.

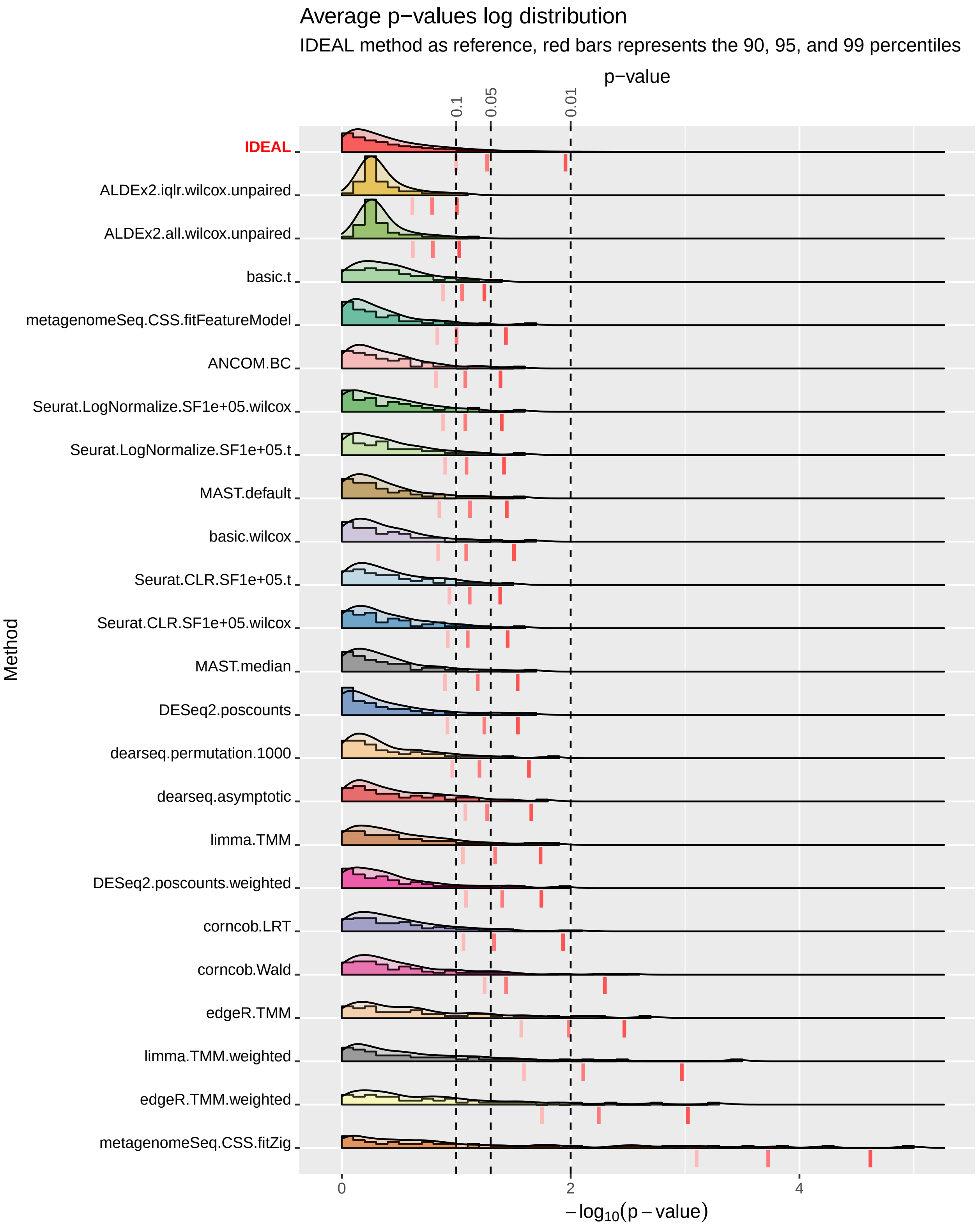

Figure 14: -log10(average p-value) plot ('average' refers to the average p-value computed for each quantile across mocks comparisons). Negative logarithm distribution of average p-values. Red-shaded vertical bars represent the 90, 95, and 99 percentiles for the negative log distribution of average p-values for each method. They should align with the dotted lines which represent the percentiles of the IDEAL distribution.

In the figure 13 and 14, the $-\log_{10}(p - value)$ IDEAL distribution is reported in red color as the first method. To highlight tail's behaviors, 3 percentiles (0.9, 0.95, 0.99) are reported using red-shaded vertical segments for each method. If the method's distribution of negative log-transformed p-values or average p-values is still uniform in the 3 selected quantiles of the tail, the 3 red vertical segments will align to the respective dotted line. Methods are ordered using the distances between the observed quantiles and the ideal ones. Usually, when a method has its red segments to the left of the IDEAL's ones is conservative (*e.g.*, `ALDEx2.iqlr.wilcox.unpaired` and `MAST.default`). Indeed, for those methods, little p-values are fewer than expected. On the contrary, methods with red segments to the right of the IDEAL's ones are liberal (*e.g.*, `edgeR.TMM`). Mixed results could be present: a method that has a lower quantile for one threshold and higher quantiles for the others (*e.g.*, `limma.TMM`).

4.6 Discussion about TIEC

Putting all the previous graphical representations together gives a general overview of methods' ability to control FPs and p-values distribution under the null hypothesis (*i.e.* no differential abundance). It is clear that only methods that produce p-values can be included in this analysis. While figures 10 and 11 have a main exploratory scope regarding the p-values distribution based on quantile-quantile comparison, figures 8, 12, and 14 are able to rank methods according to False Positive Rate, uniformity of p-values distribution, and departure from uniformity in the tail. The latter graphical representations could be used as a first tool to establish which DA method to consider for further analyses and which DA methods to exclude. Finally, the figure 9 can be used to assess FDR control under the null scenario. This exposes the problem of a few extremely small p-values among a collection that looks roughly uniform. If that is the case, the Type I error would be under control but the FDR would be inflated.

5 Concordance

Assumption: Applying different methods to the same data may produce different results.

Questions: How much do the methods agree with each other? How much does a method agree with itself?

5.1 Concordance structure

To measure the ability of each method to produce replicable results from a dataset with two or more groups:

1. samples are divided to obtain the Subset1 and Subset2 datasets using the `createSplits()` function;
2. DA methods are run on both subsets using the `runSplits()` function;
3. the Concordance At the Top metric (CAT) between the lists of p-values is computed to obtain the Between Methods Concordance (BMC) and the Within Method Concordance (WMC);
4. steps 1-3 are repeated many times ($N = 10$, but at least 100 are suggested) and the results are averaged using the `createConcordance()` function.

The relationships between the functions used in this section are explained by the diagram in Figure 15.

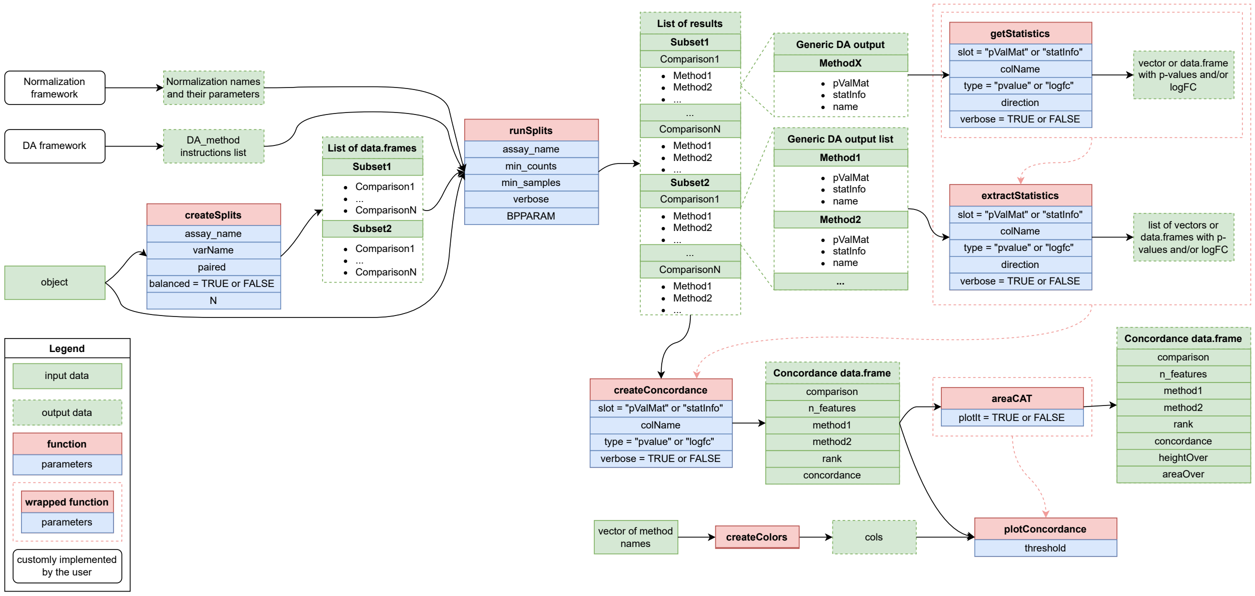

Figure 15: Concordance diagram.

5.2 Split datasets

Using the `createSplits()` function, the *ps_plaque_16S* dataset is randomly divided by half. In this dataset, samples are paired: 1 sample for supragingival plaque and 1 sample for subgingival plaque are considered for each subject. The *paired* parameter is passed to the method (it contains the name of the variable which describes the subject IDs) so the paired samples are inside the same split. In this specific case, the two groups of samples are balanced between conditions, reflecting the starting dataset. However, if the starting dataset had been unbalanced, the *balanced* option would have allowed to keep the two splits unbalanced or not.

```
set.seed(123)

# Make sure that groups and subject IDs are factors
sample_data(ps_plaque_16S)$HMP_BODY_SUBSITE <-
    factor(sample_data(ps_plaque_16S)$HMP_BODY_SUBSITE)
```

```r
sample_data(ps_plaque_16S)$RSID <-
    factor(sample_data(ps_plaque_16S)$RSID)

my_splits <- createSplits(
  object = ps_plaque_16S,
  varName = "HMP_BODY_SUBSITE",
  paired = "RSID",
  balanced = TRUE,
  N = 10
) # At least 100 is suggested
```

The structure produced by `createSplits()` function consists in a list of two matrices: *Subset1* and *Subset2*. Each matrix contains the randomly chosen sample IDs. The number of rows of both matrices is equal to the number of comparisons/splits (10 in this example, but at least 100 are suggested).

5.3 Set up normalizations and DA methods

For some of the methods implemented in this package it is possible to perform differential abundance testings for the repeated measurements experimental designs (*e.g.*, by adding the subject ID in the model formula of `DESeq2`).

Once again, to set the differential abundance methods to use, the `set_<name_of_the_method>()` methods can be exploited. For a faster demonstration, differential abundance methods without weighting are used:

```r
my_edgeR_noWeights <- set_edgeR(
    group_name = "HMP_BODY_SUBSITE",
    design = ~ 1 + RSID + HMP_BODY_SUBSITE,
    coef = "HMP_BODY_SUBSITESupragingival Plaque",
    norm = "TMM")

my_DESeq2_noWeights <- set_DESeq2(
    contrast = c("HMP_BODY_SUBSITE",
    "Supragingival Plaque", "Subgingival Plaque"),
    design = ~ 1 + RSID + HMP_BODY_SUBSITE,
    norm = "poscounts")

my_limma_noWeights <- set_limma(
    design = ~ 1 + RSID + HMP_BODY_SUBSITE,
    coef = "HMP_BODY_SUBSITESupragingival Plaque",
    norm = "TMM")

my_ALDEx2 <- set_ALDEx2(
    pseudo_count = FALSE,
    design = "HMP_BODY_SUBSITE",
    mc.samples = 128,
    test = "wilcox",
    paired.test = TRUE,
    denom = "all",
    contrast = c("HMP_BODY_SUBSITE", "Supragingival Plaque", "Subgingival Plaque"))

my_MAST <- set_MAST(
    pseudo_count = FALSE,
    rescale = "median",
    design = "~ 1 + RSID + HMP_BODY_SUBSITE",
    coefficient = "HMP_BODY_SUBSITESupragingival Plaque")
```

```r
my_dearseq <- set_dearseq(
    pseudo_count = FALSE,
    covariates = NULL,
    variables2test = "HMP_BODY_SUBSITE",
    sample_group = "RSID",
    test = "asymptotic",
    preprocessed = FALSE)

# Very time consuming
my_ANCOM <- set_ANCOM(
    pseudo_count = FALSE,
    adj_formula = NULL,
    rand_formula = "~ 1 | RSID",
    contrast = c("HMP_BODY_SUBSITE",
    "Supragingival Plaque", "Subgingival Plaque"),
    BC = FALSE)

my_methods_noWeights <- c(
    my_edgeR_noWeights,
    my_DESeq2_noWeights,
    my_limma_noWeights,
    my_ALDEx2,
    my_MAST,
    my_dearseq,
    my_ANCOM)
```

Similarly, to set the normalization methods, the `setNormalizations()` function can be used. In this case it has already been set up for the TIEC analysis:

```r
str(my_normalizations)
```

```
## List of 3
##  $ norm_edgeR :List of 2
##   ..$ fun   : chr "norm_edgeR"
##   ..$ method: chr "TMM"
##  $ norm_DESeq2:List of 2
##   ..$ fun   : chr "norm_DESeq2"
##   ..$ method: chr "poscounts"
##  $ norm_CSS   :List of 2
##   ..$ fun   : chr "norm_CSS"
##   ..$ method: chr "CSS"
```

The `runSplits()` function generates the subsets and performs DA analysis on the features with at least 1 ($min\_counts > 0$) count in more than 2 samples ($min\_samples > 2$). As for `runMocks()` function, also `runSplits()` supports parallel computing.

```r
# Set the parallel framework
# Remember that ANCOMBC based methods are only compatible with SerialParam()
bpparam <- BiocParallel::SerialParam()

# Make sure the subject ID variable is a factor
phyloseq::sample_data(ps_plaque_16S)[, "RSID"] <- as.factor(
    phyloseq::sample_data(ps_plaque_16S)[["RSID"]])

Plaque_16S_splitsDA <- runSplits(
```

```
    split_list = my_splits,
    method_list = my_methods_noWeights,
    normalization_list = my_normalizations,
    object = ps_plaque_16S,
    min_counts = 0, min_samples = 2,
    verbose = FALSE,
    BPPARAM = bpparam)
```

The structure of the output in this example is the following:

- *Subset1* and *Subset2* on the first level, which contains:
  - *Comparison1* to *Comparison10* output lists on the second level:
    - results of 7 methods on the third level: `edgeR` with *TMM* scaling factors, `DESeq2` with *poscounts* normalization factors, `limma-voom` with *TMM* scaling factors (all the 3 previous methods have the Subject identifier in the design formula), `ALDEx2` with paired *wilcox* test and denom equals to *all*, `MAST` with *median* scaling and the subject identifier in the design formula, `dearseq` for repeated measures with *asymptotic* test, and `ANCOM` without bias correction. Their outputs are organized as always:
      - *pValMat* which contains the matrix of raw p-values and adjusted p-values in *rawP* and *adjP* columns respectively;
      - `statInfo` which contains the matrix of summary statistics for each feature, such as the *logFC*, standard errors, test statistics and so on;
      - *dispEsts* which contains the dispersion estimates for methods like *edgeR* and *DESeq2*;
      - *name* which contains the complete name of the used method.

5.3.1 Add a new DA method later in the analysis

Again, it may happen that at a later time the user wants to add to the results already obtained, the results of another group of methods. First of all, the same splits and the same object must be used to obtain the new results:

```
my_basic <- set_basic(
    pseudo_count = FALSE,
    contrast = c("HMP_BODY_SUBSITE",
    "Supragingival Plaque", "Subgingival Plaque"),
    test = "wilcox",
    paired = TRUE)

Plaque_16S_splitsDA_basic <- runSplits(
    split_list = my_splits,
    method_list = my_basic,
    normalization_list = NULL,
    object = ps_plaque_16S,
    min_counts = 0, min_samples = 2,
    verbose = FALSE)
```

To put everything together, two nested `mapply`s can be used to exploit the output structures:

```
Plaque_16S_splitsDA_all <- mapply(
    Plaque_16S_splitsDA, # List of old results
    Plaque_16S_splitsDA_basic, # List of new results
    FUN = function(subset_old, subset_new){
        mapply(
```

```
            subset_old,
            subset_new,
            FUN = function(old, new){
                return(c(old, new))
        }, SIMPLIFY = FALSE)
    }, SIMPLIFY = FALSE)
```

5.4 Comparing the concordances

For each pair of methods the concordance is computed by the `createConcordance()` function. It produces a long format data frame object with several columns:

- *comparison* which indicates the comparison number;
- *n_features* which indicates the total number of taxa in the comparison dataset;
- name of *method1*;
- name of *method2*;
- *rank*;
- *concordance* which is defined as the cardinality of the intersection of the top *rank* elements of each list, divided by *rank*, *i.e.*, $\frac{L_{1:rank} \bigcap M_{1:rank}}{rank}$, where L and M represent the lists of p-values of *method1* and *method2* respectively. A noise value ($< 10^{-10}$) is added to each p-value (or statistic) in order to avoid duplicated values which could not be ordered.

```
concordance <- createConcordance(
    object = Plaque_16S_splitsDA_all,
    slot = "pValMat",
    colName = "rawP",
    type = "pvalue"
)

head(concordance)
```

```
##   rank concordance n_features   method1   method2  comparison
## 1    1   0.0000000         47 edgeR.TMM edgeR.TMM Comparison1
## 2    2   0.5000000         47 edgeR.TMM edgeR.TMM Comparison1
## 3    3   0.6666667         47 edgeR.TMM edgeR.TMM Comparison1
## 4    4   1.0000000         47 edgeR.TMM edgeR.TMM Comparison1
## 5    5   0.8000000         47 edgeR.TMM edgeR.TMM Comparison1
## 6    6   0.6666667         47 edgeR.TMM edgeR.TMM Comparison1
```

The `createConcordance()` method is very flexible. In the example below the concordances are built using the log fold changes or other statistics instead of the p-values. To do so, it is necessary to know the column names generated by each differential abundance method in the *statInfo* matrix.

Firstly, the method order is extracted using the *name* slot:

```
names(Plaque_16S_splitsDA_all$Subset1$Comparison1)
```

```
## [1] "edgeR.TMM"                     "DESeq2.poscounts"
## [3] "limma.TMM"                     "ALDEx2.all.wilcox.paired"
## [5] "MAST.median"                   "dearseq.repeated.asymptotic"
## [7] "ANCOM"                         "basic.wilcox.paired"
```

Then, the column names of the *statInfo* slot are investigated:

```
cat("edgeR.TMM", "\n")
```

```
## edgeR.TMM
```

```r
names(Plaque_16S_splitsDA_all$Subset1$Comparison1$edgeR.TMM$statInfo)
```

```
## [1] "logFC"  "logCPM" "F"      "PValue"
```

```r
cat("DESeq2.poscounts", "\n")
```

```
## DESeq2.poscounts
```

```r
names(Plaque_16S_splitsDA_all$Subset1$Comparison1$DESeq2.poscounts$statInfo)
```

```
## [1] "baseMean"       "log2FoldChange" "lfcSE"          "stat"
## [5] "pvalue"         "padj"
```

```r
cat("limma.TMM", "\n")
```

```
## limma.TMM
```

```r
names(Plaque_16S_splitsDA_all$Subset1$Comparison1$limma.TMM$statInfo)
```

```
## [1] "logFC"     "AveExpr"   "t"         "P.Value"   "adj.P.Val" "B"
```

```r
cat("ALDEx2.all.wilcox.paired", "\n")
```

```
## ALDEx2.all.wilcox.paired
```

```r
names(Plaque_16S_splitsDA_all$Subset1$Comparison1$ALDEx2.all.wilcox.paired$
    statInfo)
```

```
##  [1] "rab.all"                      "rab.win.Subgingival Plaque"
##  [3] "rab.win.Supragingival Plaque" "diff.btw"
##  [5] "diff.win"                     "effect"
##  [7] "overlap"                      "we.ep"
##  [9] "we.eBH"                       "wi.ep"
## [11] "wi.eBH"
```

```r
cat("MAST.median", "\n")
```

```
## MAST.median
```

```r
names(Plaque_16S_splitsDA_all$Subset1$Comparison1$MAST.median$statInfo)
```

```
## [1] "logFC"    "logFC.lo" "logFC.hi" "rawP"     "adjP"
```

```r
cat("dearseq.repeated.asymptotic", "\n")
```

```
## dearseq.repeated.asymptotic
```

```r
names(Plaque_16S_splitsDA_all$Subset1$Comparison1$dearseq.repeated.asymptotic$
    statInfo)
```

```
## [1] "rawP" "adjP"
```

```r
cat("ANCOM", "\n")
```

```
## ANCOM
```

```r
names(Plaque_16S_splitsDA_all$Subset1$Comparison1$ANCOM$statInfo)
```

```
## [1] "taxon"         "W"             "detected_0.9" "detected_0.8" "detected_0.7"
## [6] "detected_0.6"
```

```r
cat("basic.wilcox.paired", "\n")
```

```
## basic.wilcox.paired
```

```
names(Plaque_16S_splitsDA_all$Subset1$Comparison1$basic.wilcox.paired$statInfo)
```

```
## [1] "taxon"     "statistic" "pvalue"    "logFC"
```

All methods, except for `DESeq2`, `ALDEx2`, `dearseq`, and `ANCOM`, contain the log fold change values in the *logFC* column of `statInfo` matrix. Knowing this, the alternative concordance data frame can be built using:

```
concordance_alternative <- createConcordance(
    object = Plaque_16S_splitsDA_all,
    slot = "statInfo",
    colName = c("logFC", "log2FoldChange", "logFC", "effect", "logFC", "rawP",
        "W", "logFC"),
    type = c("logfc", "logfc", "logfc", "logfc", "logfc", "pvalue", "logfc",
        "logfc")
)
```

5.5 Visualization

Starting from the table of concordances, the `plotConcordance()` function can produce 2 graphical results visible in Figure 16:

- the dendrogram of methods, clustered by the area over the concordance bisector in *concordanceDendrogram* slot;
- the heatmap of the between and within method concordances in *concordanceHeatmap* slot. For each tile of the symmetric heatmap, which corresponds to a pair of methods, the concordance from rank 1 to a threshold rank is drawn.

The area between the curve and the bisector is colored to highlight concordant methods (blue) and non-concordant ones (red). The two graphical results should be drawn together for the best experience.

```
pC <- plotConcordance(
    concordance = concordance,
    threshold = 30)

cowplot::plot_grid(
    plotlist = pC,
    ncol = 2,
    align = "h",
    axis = "tb",
    rel_widths = c(1, 3))
```

The WMC and BMC from rank 1 to rank 30 are reported in Figure 16. More than 40 (use `table(concordance$rank)` to find out) is the maximum rank obtained by all split comparisons, *i.e.* the number of taxa for which all methods calculated p-values (in all comparisons). However, a custom threshold of 30 was supplied.

It is common that WMC values (in red rectangles) are lower than BMC ones. Indeed, BMC is computed between different methods on the same data, while WMC is computed for a single method, run in different datasets (some taxa are dataset-specific).

`limma.TMM` and `edgeR.TMM` methods show the highest BMC values but they are also concordant with `dearseq.repeated.asymptotic` and `basic.wilcox.paired`. Differently, `DESeq2.poscounts` and `MAST.median` are not concordant with the other methods. `ANCOM` shows a completely random pattern (the lines are always close to the dashed line) because all the chosen statistics are equal to 1 and they can't be ordered.

Regarding the WMC, `ALDEx2.all.wilcox.paired` has the highest value while `MAST.median` has the lowest

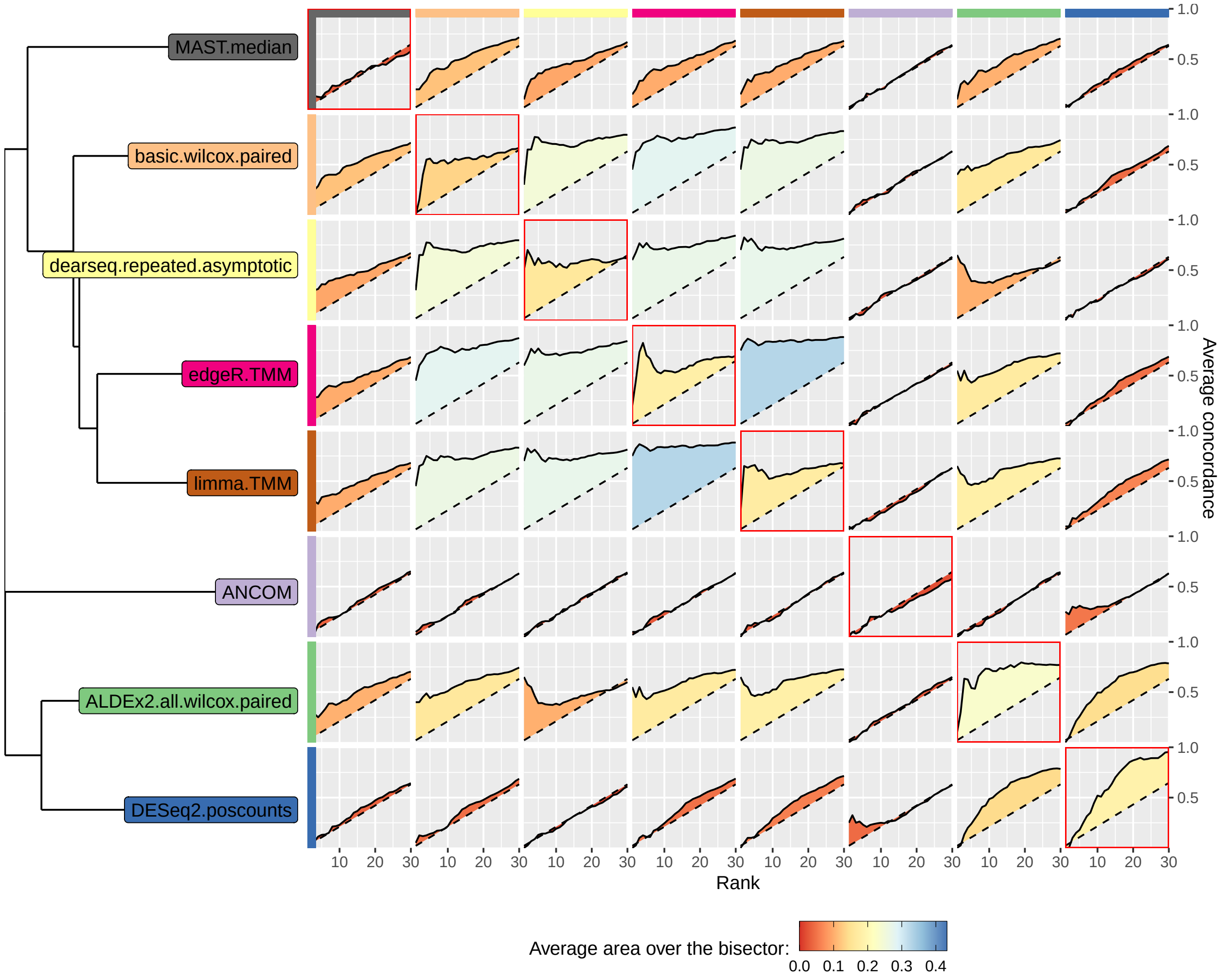

Figure 16: BMC and WMC plot. Between-method concordance (BMC) and within-method concordance (WMC) (main diagonal) averaged values from rank 1 to 30.

values, maybe because it has not been implemented properly for repeated measure designs. Other methods have comparable WMC values.

5.6 Discussion about Concordance

Random splits allow to evaluate concordance between methods and within a method. These analyses do not assess the correctness of the discoveries. Even the method with the highest WMC could nonetheless consistently identify false positive DA taxa. For this reason, the concordance analysis framework should be used as a tool to detect groups of similar methods.

6 Enrichment analysis

Assumption: Previous analyses did not assess the correctness of the discoveries.

Question: If some prior knowledge about the experiment is available, would the findings be coherent with that knowledge?

6.1 Enrichment structure

While the lack of ground truth makes it challenging to assess the validity of DA results in real data, enrichment analysis can provide an alternative solution to rank methods in terms of their ability to identify, as significant, taxa that are known to be differentially abundant between two groups. To run methods, the `runDA()` function is used. Leveraging the prior knowledge (if present), the correctness of the findings is checked using the `createEnrichment()` and `createPositives()` functions. Many graphical outputs are available through the `plotContingency()`, `plotEnrichment()`, `plotMutualFindings()`, and `plotPositives()` functions.

The relationships between the functions used in this section are explained by the diagram in Figure 17.

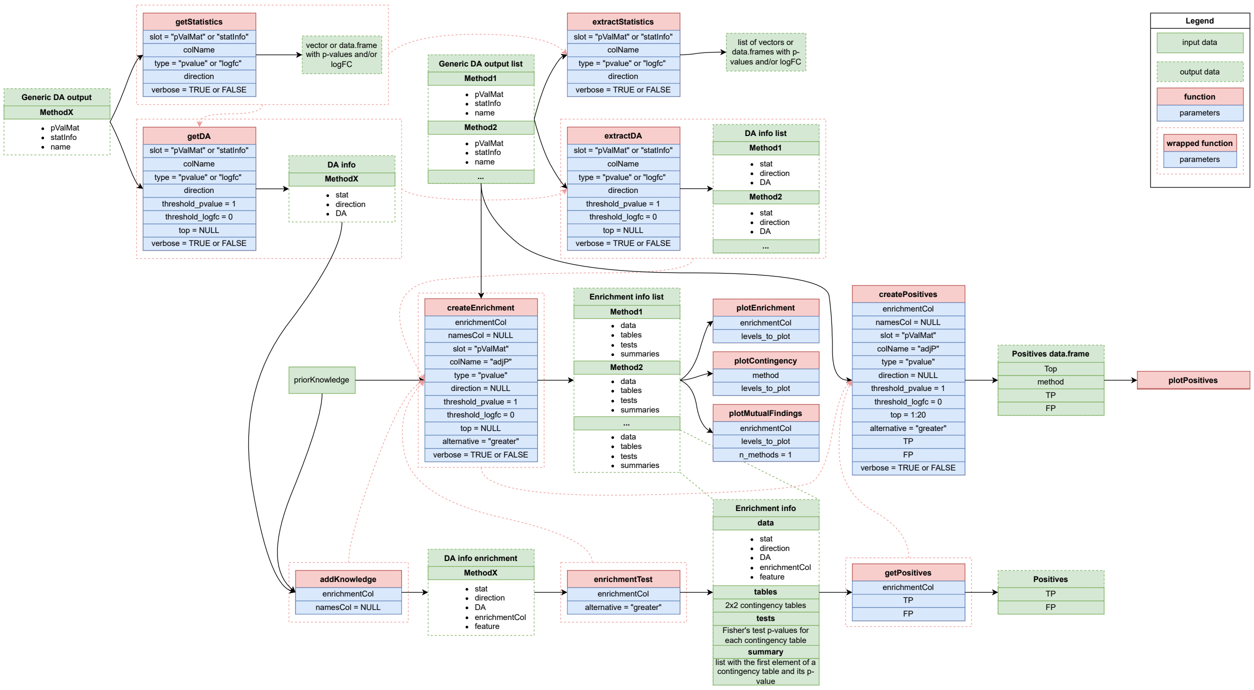

Figure 17: Enrichment analysis diagram.

6.2 *A priori* knowledge

Here, we leveraged the peculiar environment of the gingival site (Thurnheer *et al.*, 2016):

- the **supragingival biofilm** is directly exposed to the open atmosphere of the oral cavity, favoring the growth of aerobic species;
- in the **subgingival biofilm**, the atmospheric conditions gradually become strict anaerobic, favoring the growth of anaerobic species.

From the comparison of the two sites, an abundance of **aerobic microbes in the supragingival plaque** and of **anaerobic bacteria in the subgingival plaque** is expected. DA analysis should reflect this difference by finding an enrichment of aerobic (anaerobic) bacteria among the DA taxa with a positive (negative) log-fold-change.

Firstly, the microbial metabolism information is necessary. These data comes from (Beghini *et al.*, 2019) research article's github repository (https://github.com/waldronlab/nychanesmicrobiome), but they can be loaded using `data("microbial_metabolism")`:

```
data("microbial_metabolism")
head(microbial_metabolism)
```

```
##                Genus        Type
## 1     Acholeplasma F Anaerobic
## 2 Actinomycetaceae F Anaerobic
## 3    Aeriscardovia     Aerobic
## 4       Aerococcus F Anaerobic
## 5  Aggregatibacter F Anaerobic
## 6    Alloscardovia   Anaerobic
```

The microbial genus and its type of metabolism are specified in the first and second column respectively. To match each taxon of the phyloseq object to its type of metabolism the next chunk of code can be used:

```
# Extract genera from the phyloseq tax_table slot
genera <- tax_table(ps_plaque_16S)[, "GENUS"]

# Genera as rownames of microbial_metabolism data.frame
rownames(microbial_metabolism) <- microbial_metabolism$Genus

# Match OTUs to their metabolism
priorInfo <- data.frame(genera, "Type" =  microbial_metabolism[genera, "Type"])
unknown_metabolism <- is.na(priorInfo$Type)
priorInfo[unknown_metabolism, "Type"] <- "Unknown"

# Relabel 'F Anaerobic' to 'F_Anaerobic' to remove space
priorInfo$Type <- factor(priorInfo$Type,
    levels = c("Aerobic","Anaerobic","F Anaerobic","Unknown"),
    labels = c("Aerobic","Anaerobic","F_Anaerobic","Unknown"))

# Add a more informative names column
priorInfo[, "newNames"] <- paste0(rownames(priorInfo), "|",
    priorInfo[, "GENUS"])
```

6.3 Set up normalizations and DA methods

Both the normalization/scaling factors and the DA methods' instructions are available since the dataset is the same used in the previous section.

In concordance analysis, normalizations factor were added inside the `runSlits()` function, so the original object *ps_plaque_16S* does not contain the values. The normalization/scaling factors are added to the object:

```
ps_plaque_16S <- runNormalizations(my_normalizations, object = ps_plaque_16S)
```

A simple filter to remove rare taxa is applied:

```
ps_plaque_16S <- phyloseq::filter_taxa(physeq = ps_plaque_16S,
    flist = function(x) sum(x > 0) >= 3, prune = TRUE)
ps_plaque_16S
```

```
## phyloseq-class experiment-level object
## otu_table()   OTU Table:         [ 58 taxa and 60 samples ]
## sample_data() Sample Data:       [ 60 samples by 10 sample variables ]
## tax_table()   Taxonomy Table:    [ 58 taxa by 6 taxonomic ranks ]
```

```
## phy_tree()    Phylogenetic Tree: [ 58 tips and 57 internal nodes ]
```

Differently from the Type I Error Control and Concordance analyses, the enrichment analysis rely on a single `phyloseq` or `TreeSummarizedExperiment` object (no mocks, no splits, no comparisons). For this reason many methods can be assessed without computational trade-offs (*e.g.*, `ANCOM` without sampling fraction bias correction and methods which use *ZINB* weights).

The observational weights are computed:

```
plaque_weights <- weights_ZINB(object = ps_plaque_16S, design = ~ 1,
    zeroinflation = TRUE)
```

The existing instructions are concatenated with the instructions of methods which use observational weights:

```
my_edgeR <- set_edgeR(
    group_name = "HMP_BODY_SUBSITE",
    design = ~ 1 + RSID + HMP_BODY_SUBSITE,
    coef = "HMP_BODY_SUBSITESupragingival Plaque",
    norm = "TMM",
    weights_logical = TRUE)

my_DESeq2 <- set_DESeq2(
    contrast = c("HMP_BODY_SUBSITE",
    "Supragingival Plaque", "Subgingival Plaque"),
    design = ~ 0 + RSID + HMP_BODY_SUBSITE,
    norm = "poscounts",
    weights_logical = TRUE)

my_limma <- set_limma(
    design = ~ 1 + RSID + HMP_BODY_SUBSITE,
    coef = "HMP_BODY_SUBSITESupragingival Plaque",
    norm = "TMM",
    weights_logical = TRUE)

my_methods <- c(my_methods_noWeights, my_edgeR, my_DESeq2, my_limma)
```

All the ingredients are ready to run DA methods:

```
Plaque_16S_DA <- runDA(method_list = my_methods,
    object = ps_plaque_16S, weights = plaque_weights, verbose = FALSE)
```

6.4 Testing the enrichment

`Plaque_16_DA` object contains the results for 10 methods. In order to extract p-values, the optional direction of DA (DA vs non-DA, or UP Abundant vs DOWN Abundant), and to add any *a priori* information, the `createEnrichment()` function can be used.

In the *direction* argument, which is set to *NULL* by default, the column name containing the direction (*e.g.*, *logfc*, *logFC*, *logFoldChange*. . .) of each method's *statInfo* matrix can be supplied.

Firstly, the order of methods is investigated:

```
names(Plaque_16S_DA)
```

```
##  [1] "edgeR.TMM"                    "DESeq2.poscounts"
##  [3] "limma.TMM"                    "ALDEx2.all.wilcox.paired"
##  [5] "MAST.median"                  "dearseq.repeated.asymptotic"
##  [7] "ANCOM"                        "edgeR.TMM.weighted"
##  [9] "DESeq2.poscounts.weighted"    "limma.TMM.weighted"
```

Following the methods' order, the *direction* parameter is supplied together with other parameters:

- *threshold_pvalue*, *threshold_logfc*, and *top* (optional), to set differential abundance thresholds;
- *slot*, *colName*, and *type*, which specify where to apply the above thresholds;
- *priorKnowledge*, *enrichmentCol*, and *namesCol*, to add enrichment information to DA analysis;

The `createEnrichment()` function, with the direction parameter for all method except `ANCOM` and `dearseq` (the first has the W statistic which is only positive, while the second has only p-values), is used:

```
enrichment <- createEnrichment(
    object = Plaque_16S_DA[-c(6:7)],
    priorKnowledge = priorInfo,
    enrichmentCol = "Type",
    namesCol = "newNames",
    slot = "pValMat",
    colName = "adjP",
    type = "pvalue",
    direction = c(
        "logFC", # edgeR
        "log2FoldChange", # DEseq2
        "logFC", # limma
        "effect", # ALDEx2
        "logFC", # MAST
        "logFC", # edgeR with weights
        "log2FoldChange", # DESeq2 with weights
        "logFC"), # limma with weights
    threshold_pvalue = 0.1,
    threshold_logfc = 0,
    top = NULL,
    alternative = "greater",
    verbose = TRUE
)
```

The produced *enrichment* object consists in a list of elements as long as the number of tested methods:

- the *data* slot contains information for each feature. P-values, adjusted p-values (or other statistics) in *stats* column, log fold changes (or other statistics, if specified) in *direction* column, differential abundance information in the *DA* column (according to the thresholds), the variable of interest for the enrichment analysis, and the name of the feature in the *feature* column;
- in the *tables* slot a maximum of *2 x (levels of enrichment variable)* contingency tables (2x2) are present;
- in the *tests* slot, the list of Fisher exact tests produced by the `fisher.test()` function are saved for each contingency table;
- in the *summaries* slot, the first elements of the contingency tables and the respective p-values are collected for graphical purposes.

6.5 Visualization

6.5.1 Contingency tables

Considering one of the methods, `DESeq2.poscounts`, 8 contingency tables are obtained. Both *UP Abundant* and *DOWN Abundant* taxa are found and the enrichment variable has *Aerobic*, *Anaerobic*, *F_Anaerobic*, and *Unknown* levels. For each level, 2 contingency tables could be built: one for DOWN Abundant vs non-DOWN Abundant features and one for *UP Abundant* vs *non-UP Abundant* features. The enrichment is tested using Fisher exact test. The `plotContingency()` function summarize all these information (Figure 18).

```
plotContingency(enrichment = enrichment,
    levels_to_plot = c("Aerobic", "Anaerobic"),
    method = "DESeq2.poscounts")
```

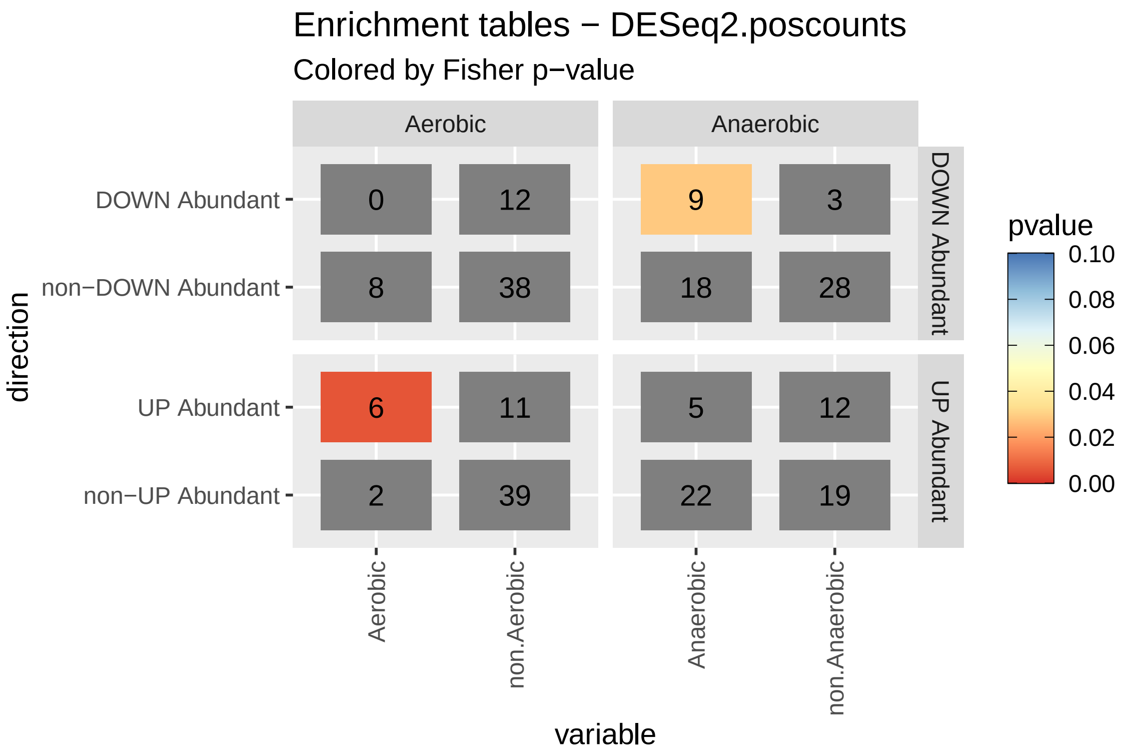

Figure 18: Contingency tables plot. Contingency tables for Aerobic and Anaerobic taxa found as differentially abundant by DESeq2.poscounts DA method. Fisher exact test has been performed on each contingency table. If the enrichment is signficantly present, the corresponding cell will be highlighted.

6.5.2 Enrichment plot

To summarize enrichment analysis for all the methods simultaneously, the `plotEnrichment()` function can be used. Only *Aerobic* and *Anaerobic* levels are plotted in Figure 19.

```
plotEnrichment(enrichment = enrichment, enrichmentCol = "Type",
    levels_to_plot = c("Aerobic", "Anaerobic"))
```

Since *Subgingival Plaque* is the reference level for each method, the coefficients extracted from the methods are referred to the *Supragingival Plaque* class. Six out of eight methods identify, as expected, a statistically significant ($0.001 < p \leq 0.05$) amount of DOWN Abundant Anaerobic features in Supragingival Plaque (Figure 19). Moreover, all of them find an enriched amount of UP Abundant Aerobic genera in Supragingival Plaque. Unexpectedly, both `DESeq2.poscounts` and `DESeq2.poscounts.weighted` find many Anaerobic genera as UP Abundant, they could be FPs.

6.5.3 Mutual Findings

To investigate the DA features, the `plotMutualFindings()` function can be used (Figure 20). While *levels_to_plot* argument allows to choose which levels of the enrichment variable to plot, *n_methods* argument allows to extract only features which are mutually found as DA by more than 1 method.

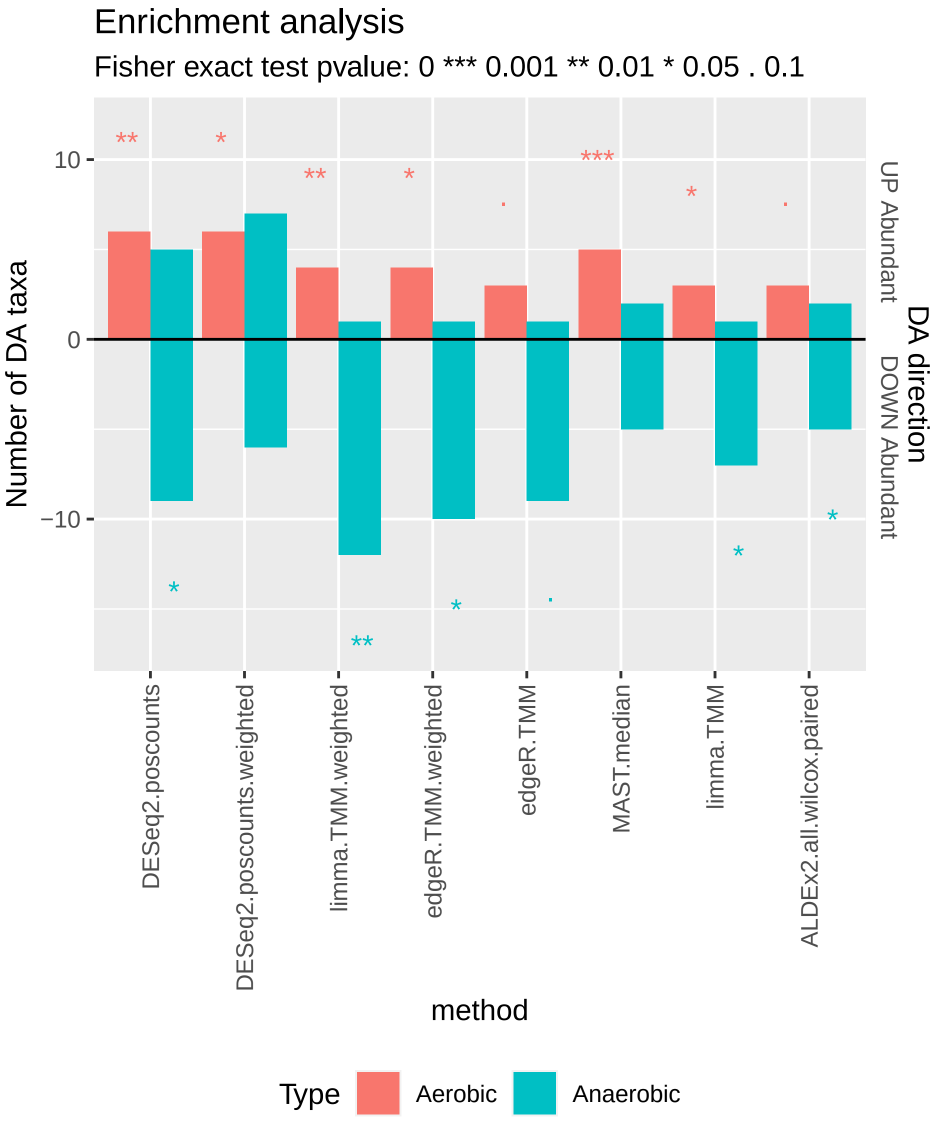

Figure 19: Enrichment plot. Number of differentially abundant features, colored by aerobic or anaerobic metabolism, and directed according to differential abundance direction (UP or DOWN abundant).

```r
plotMutualFindings(enrichment, enrichmentCol = "Type",
    levels_to_plot = c("Aerobic", "Anaerobic"), n_methods = 1)
```

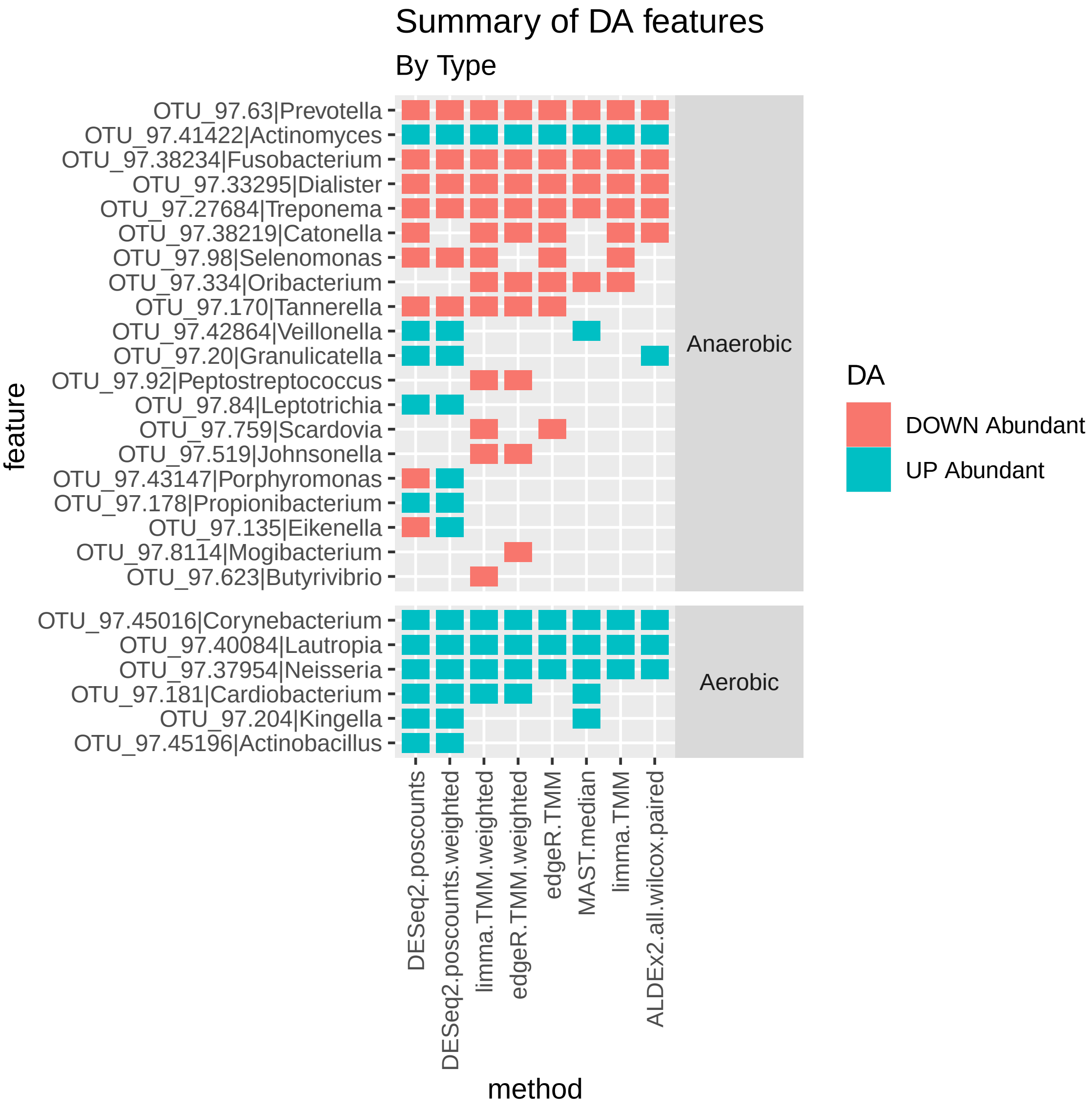

Figure 20: Mutual Findings plot. Number of differentially abundant features mutually found by 1 or more methods, colored by the differential abundance direction and separated by aerobic and anaerobic metabolism.

In this example (Figure 20), many Anaerobic genera and 6 Aerobic genera are found as DA by more than 1 method simultaneously. Among them, all methods find *Prevotella*, *Treponema*, *Fusobacterium*, and *Dialister* genera DOWN Abundant in Supragingival Plaque, while the *Actinomyces* genus UP Abundant, even if it has an aerobic metabolism. Similarly, all methods find *Corynebacterium*, *Leutropia,* and *Neisseria* aerobic genera UP abundant in Supragingival Plaque.

6.6 True and False Positives

To evaluate the overall performances a statistic based on the difference between putative True Positives (TP) and the putative False Positives (FP) is used. To build the matrix to plot, the `createPositives()` can be used. In details, the correctness of the DA features is evaluated comparing the direction of the top ranked

features to the expected direction supplied by the user in the *TP* and *FP* lists. The procedure is performed for several thresholds of *top* parameter in order to observe a trend, if present:

```
positives <- createPositives(
    object = Plaque_16S_DA[-c(6,7)],
    priorKnowledge = priorInfo,
    enrichmentCol = "Type",
    namesCol = "newNames",
    slot = "pValMat",
    colName = "rawP",
    type = "pvalue",
    direction = c(
        "logFC", # edgeR
        "log2FoldChange", # DEseq2
        "logFC", # limma
        "effect", # ALDEx2
        "logFC", # MAST
        "logFC", # edgeR with weights
        "log2FoldChange", # DESeq2 with weights
        "logFC"), # limma with weights
    threshold_pvalue = 0.1,
    threshold_logfc = 0,
    top = seq.int(from = 0, to = 30, by = 3),
    alternative = "greater",
    verbose = FALSE,
    TP = list(c("DOWN Abundant", "Anaerobic"), c("UP Abundant", "Aerobic")),
    FP = list(c("DOWN Abundant", "Aerobic"), c("UP Abundant", "Anaerobic"))
)
head(positives)
```

```
##   top                    method TP FP
## 1   3                 edgeR.TMM  3  0
## 2   3          DESeq2.poscounts  3  0
## 3   3                 limma.TMM  3  0
## 4   3 ALDEx2.all.wilcox.paired  1  1
## 5   3               MAST.median  3  0
## 6   3        edgeR.TMM.weighted  3  0
```

The `plotPositives()` function can be used to summarize the methods' performances (Figure 21). Higher values usually represents better performances. In our example, all methods show similar values of the statistics for the top 10 ranked features.

```
plotPositives(positives)
```

`MAST.median` and `ALDEx2.all.wilcox.paired` methods are very conservative and are located on the lower part of the Figure 21. The highest performances are of `limma.TMM.weighted` and `edgeR.TMM.weighted`. This means that their findings are in line with the *a priori* knowledge supplied by the user.

6.7 Enrichment without direction

When the user have a custom method where the direction of the differential abundance is not returned (*e.g.*, NOISeq), or when the direction of DA is not of interest, the sole information about DA and not DA feature can be used. The `createEnrichment()` function is used without the direction parameter for all methods:

```
enrichment_nodir <- createEnrichment(
    object = Plaque_16S_DA,
```

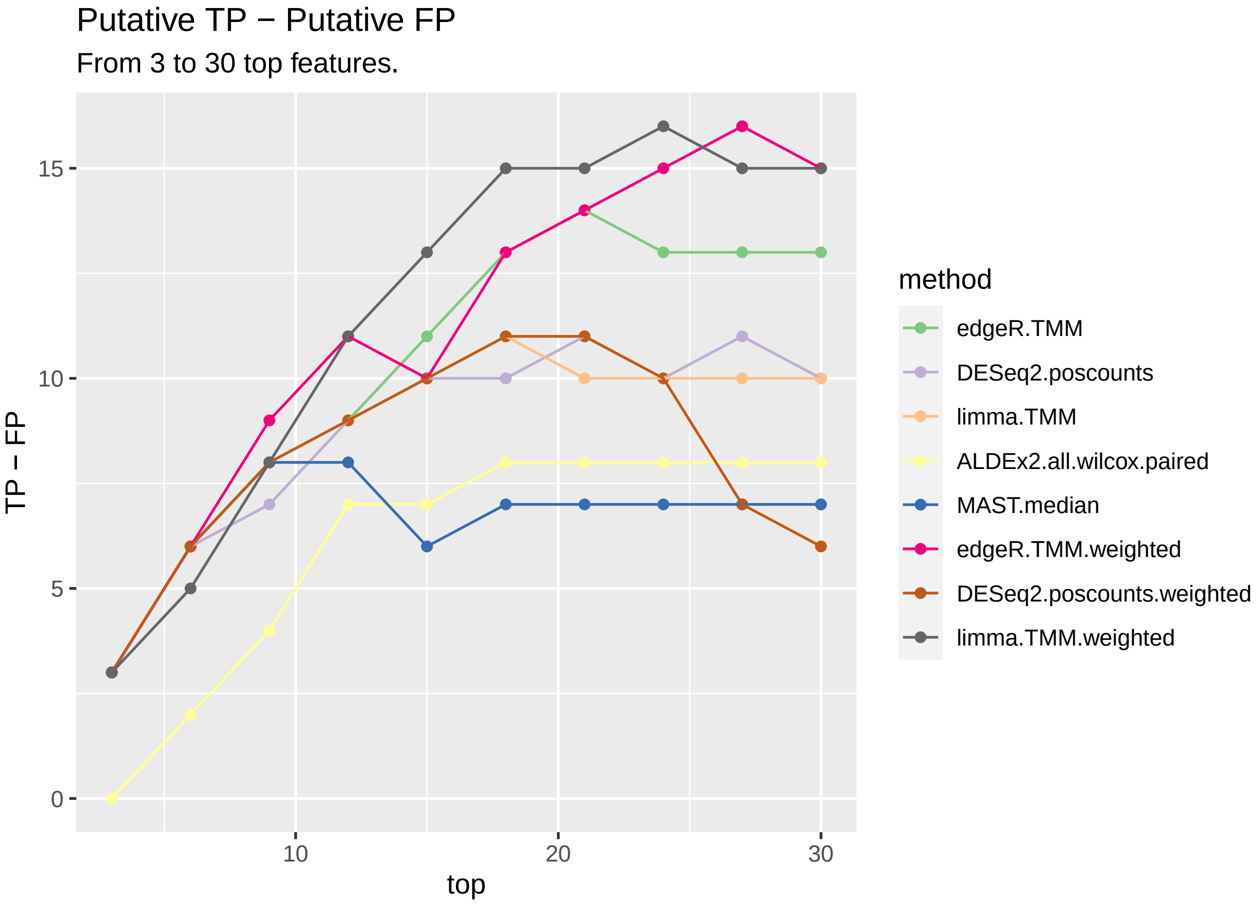

Figure 21: TP, FP differences plot. Differences between the number of True Positives and False Positives for several thresholds of the top ranked raw p-values (the top 3 lowest p-values, the top 6, 9, . . . , 30) for each method.

```
    priorKnowledge = priorInfo,
    enrichmentCol = "Type",
    namesCol = "newNames",
    slot = "pValMat",
    colName = "adjP",
    type = "pvalue",
    threshold_pvalue = c(
        0.1, 0.1, 0.1, 0.1, 0.1, 0.1, # thresholds for methods
        0.4, # ANCOM threshold on 1-W/(ntaxa-1) 0.4 = liberal
        0.1, 0.1, 0.1), # thresholds for other methods
    threshold_logfc = 0,
    top = NULL,
    alternative = "greater",
    verbose = FALSE
)
```

To summarize enrichment analysis for all the methods simultaneously, the `plotEnrichment()` function is used. All levels are plotted in Figure 22.

```
plotEnrichment(enrichment = enrichment_nodir, enrichmentCol = "Type")
```

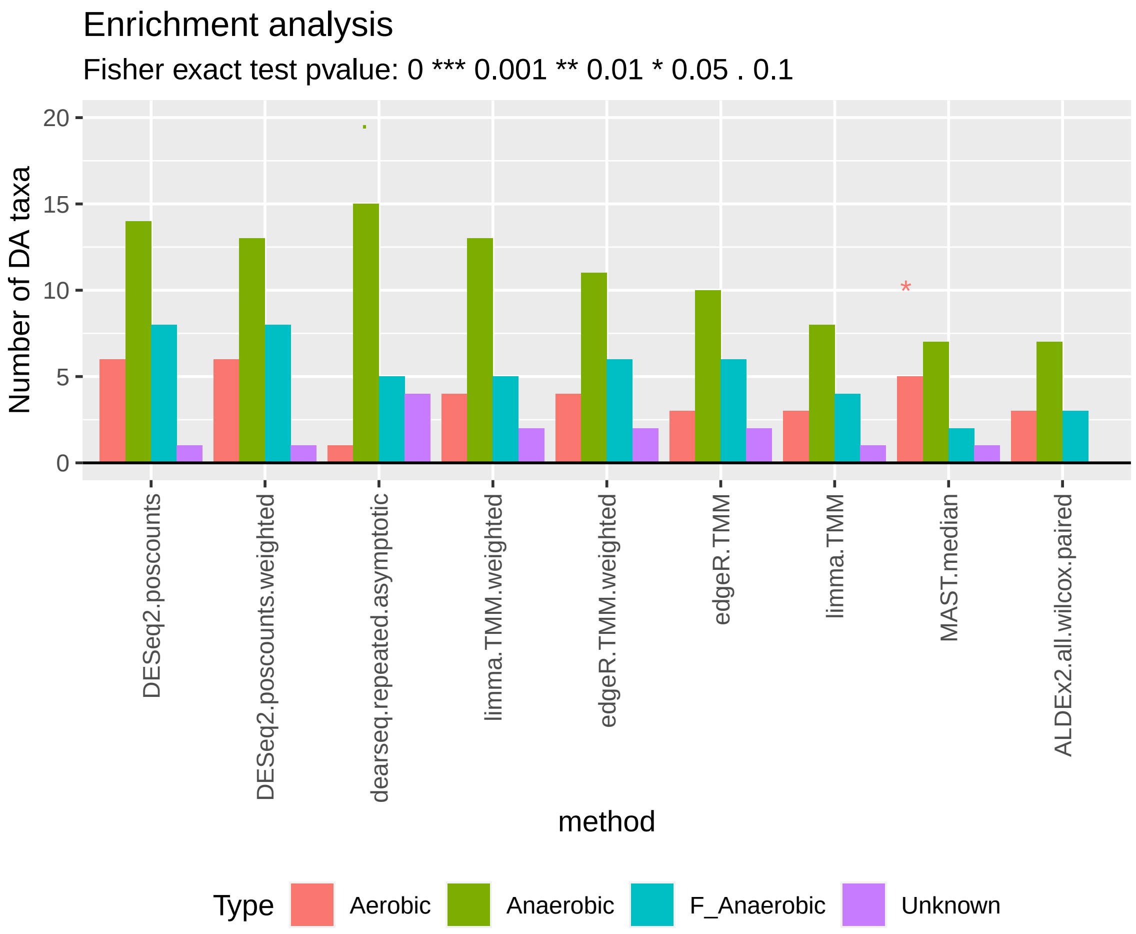

Figure 22: Enrichment plot. Number of differentially abundant features, colored by metabolism.

The highest amount of DA features belongs to the *Anaerobic* metabolism, followed by *F_Anaerobic*, and *Aerobic*. The method that finds more DA features is `DESeq2.poscounts`, while `ANCOM` is the most conservative

(even if the threshold value based on $1 - \frac{W}{ntaxa-1}$ is set at liberal value of 0.4.

As for enrichment analysis with DA direction, the `plotMutualFindings()` function can be used here too (Figure 23). While *levels_to_plot* argument allows to choose which levels of the enrichment variable to plot, *n_methods* argument allows to extract only features which are mutually found as DA by more than 1 method.

```
plotMutualFindings(enrichment_nodir, enrichmentCol = "Type",
    levels_to_plot = c("Aerobic", "Anaerobic"), n_methods = 1)
```

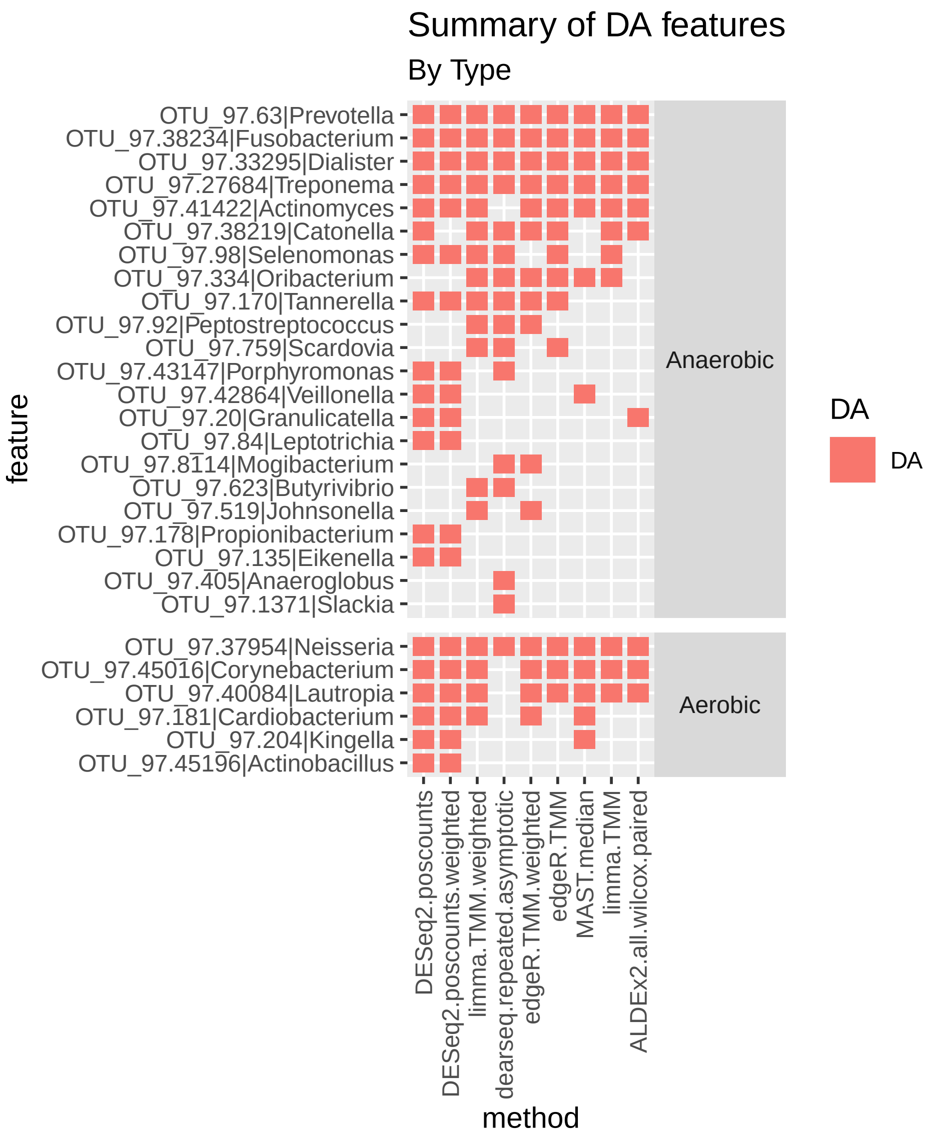

Figure 23: Mutual Findings plot. Number of differentially abundant features mutually found by 1 or more methods, separated by aerobic and anaerobic metabolism.

In this example (Figure 23), only a *Prevotella* OTU is found as DA in Supragingival Plaque by all methods (probably because `ANCOM` p-value threshold is not interpretable as the same threshold for the other methods).

6.8 Enrichment analysis for simulated data

To enlarge the scope of the enrichment analysis, simulations could be used, *e.g.* by using the user's dataset as a template to generate simulated data, in which to know the DA features and provide this information as

prior knowledge.

As an example, the `SPsimSeq` package is used (the tool to use is up to the user) to simulate only a single dataset (*n.sim = 1*) from the *ps_plaque_16S* dataset where two body sub sites are available (without considering the paired design). The data are simulated with the following properties - 100 features (*n.genes = 100*) - 50 samples (*tot.samples = 50*) - the samples are equally divided into 2 groups each with 25 samples (*group.config = c(0.5, 0.5)*) - all samples are from a single batch (*batch.config = 1*) - 20% DA features (*pDE = 0.2*) - the DA features have a log-fold-change of at least 0.5.

```
data("ps_plaque_16S")
counts_and_metadata <- get_counts_metadata(ps_plaque_16S)
plaque_counts <- counts_and_metadata[["counts"]]
plaque_metadata <- counts_and_metadata[["metadata"]]

set.seed(123)

sim_list <- SPsimSeq(
    n.sim = 1,
    s.data = plaque_counts,
    group = plaque_metadata[, "HMP_BODY_SUBSITE"],
    n.genes = 100,
    batch.config = 1,
    group.config = c(0.5, 0.5),
    tot.samples = 50,
    pDE = 0.2,
    lfc.thrld = 0.5,
    model.zero.prob = FALSE,
    result.format = "list")
```

Simulated data are organised into a `TreeSummarizedExperiment` object:

```
sim_obj <- TreeSummarizedExperiment::TreeSummarizedExperiment(
    assays = list("counts" = sim_list[[1]][["counts"]]),
    rowData = sim_list[[1]]["rowData"],
    colData = sim_list[[1]]["colData"],
)
# Group as factor
SummarizedExperiment::colData(sim_obj)[, "colData.Group"] <- as.factor(
    SummarizedExperiment::colData(sim_obj)[, "colData.Group"])
```

The *apriori* informations are readily available from the `sim_list[[1]]["rowData"]`:

```
priorInfo <- sim_list[[1]][["rowData"]]
priorInfo$Reality <- ifelse(priorInfo[, "DE.ind"], "is DA", "is not DA")
```

Once again, normalization/scaling factors are added:

```
sim_obj <- runNormalizations(
    normalization_list = my_normalizations,
    object = sim_obj,
    verbose = TRUE)
```

Rare taxa are filtered:

```
taxa_to_keep <- apply(assays(sim_obj)[["counts"]], 1,
    function(x) sum(x > 0) >= 3)
sim_obj <- sim_obj[taxa_to_keep, ]
priorInfo <- priorInfo[taxa_to_keep, ]
```

Observational weights are computed:

```
sim_weights <- weights_ZINB(
    object = sim_obj,
    design = ~ 1,
    zeroinflation = TRUE)
```

DA methods are set up. The paired design is not considered and all the methods are used. The *contrast*, *design*, *group*, *coef*, and all the other parameters involved in the experimental design definition are changed:

```
my_basic <- set_basic(pseudo_count = FALSE,
    contrast = c("colData.Group", "Supragingival Plaque",
        "Subgingival Plaque"),
    test = c("t", "wilcox"),
    paired = FALSE,
    expand = TRUE)

my_edgeR <- set_edgeR(
    pseudo_count = FALSE,
    group_name = "colData.Group",
    design = ~ colData.Group,
    robust = FALSE,
    coef = 2,
    norm = "TMM",
    weights_logical = c(TRUE, FALSE),
    expand = TRUE)

my_DESeq2 <- set_DESeq2(
    pseudo_count = FALSE,
    design = ~ colData.Group,
    contrast = c("colData.Group", "Supragingival Plaque",
        "Subgingival Plaque"),
    norm = "poscounts",
    weights_logical = c(TRUE, FALSE),
    alpha = 0.05,
    expand = TRUE)

my_limma <- set_limma(
    pseudo_count = FALSE,
    design = ~ colData.Group,
    coef = 2,
    norm = "TMM",
    weights_logical = c(FALSE, TRUE),
    expand = TRUE)

my_ALDEx2 <- set_ALDEx2(
    pseudo_count = FALSE,
    design = "colData.Group",
    mc.samples = 128,
    test = "wilcox",
    paired.test = FALSE,
    denom = c("all", "iqlr"),
    contrast = c("colData.Group", "Supragingival Plaque",
        "Subgingival Plaque"),
    expand = TRUE)
```

```r
my_metagenomeSeq <- set_metagenomeSeq(
    pseudo_count = FALSE,
    design = "~ colData.Group",
    coef = "colData.GroupSupragingival Plaque",
    norm = "CSS",
    model = "fitFeatureModel",
    expand = TRUE)

my_corncob <- set_corncob(
    pseudo_count = FALSE,
    formula = ~ colData.Group,
    formula_null = ~ 1,
    phi.formula = ~ colData.Group,
    phi.formula_null = ~ colData.Group,
    test = c("Wald", "LRT"),
    boot = FALSE,
    coefficient = "colData.GroupSupragingival Plaque")

my_MAST <- set_MAST(
    pseudo_count = FALSE,
    rescale = c("default", "median"),
    design = "~ 1 + colData.Group",
    coefficient = "colData.GroupSupragingival Plaque",
    expand = TRUE)

my_Seurat <- set_Seurat(
    pseudo_count = FALSE,
    test = c("t", "wilcox"),
    contrast = c("colData.Group", "Supragingival Plaque",
        "Subgingival Plaque"),
    norm = c("LogNormalize", "CLR"),
    scale.factor = 10^5,
    expand = TRUE
)

my_ANCOM <- set_ANCOM(
    pseudo_count = FALSE,
    fix_formula = "colData.Group",
    contrast = c("colData.Group", "Supragingival Plaque",
        "Subgingival Plaque"),
    BC = c(TRUE, FALSE),
    expand = TRUE
)

my_dearseq <- set_dearseq(
    pseudo_count = FALSE,
    covariates = NULL,
    variables2test = "colData.Group",
    preprocessed = FALSE,
    test = c("permutation", "asymptotic"),
    expand = TRUE)

my_NOISeq <- set_NOISeq(
```

```
    pseudo_count = FALSE,
    contrast = c("colData.Group", "Supragingival Plaque",
        "Subgingival Plaque"),
    norm = c("rpkm", "tmm"),
    expand = TRUE)

my_methods <- c(my_basic, my_edgeR, my_DESeq2, my_limma, my_metagenomeSeq,
    my_corncob, my_ALDEx2, my_MAST, my_Seurat, my_ANCOM, my_dearseq, my_NOISeq)
```

DA methods are run using the `runDA()` function:

```
sim_DA <- runDA(
    method_list = my_methods,
    object = sim_obj,
    weights = sim_weights,
    verbose = FALSE)
```

The `createEnrichment()` without the direction parameter for all methods is used. A 0.1 threshold for the adjusted p-values is chosen to define DA and non-DA taxa for all methods, a 0.4 threshold is used for `ANCOM` instead:

```
enrichment_nodir <- createEnrichment(
    object = sim_DA,
    priorKnowledge = priorInfo,
    enrichmentCol = "Reality",
    namesCol = NULL,
    slot = "pValMat",
    colName = "adjP",
    type = "pvalue",
    threshold_pvalue = c(
        rep(0.1,19), # adjP thresholds
        0.4, # adjP threshold for ANCOM on 1-W/(ntaxa-1)
        rep(0.1,5)), # adjP thresholds for other methods
    threshold_logfc = 0,
    top = NULL,
    alternative = "greater",
    verbose = FALSE
)
```

To summarize enrichment analysis for all the methods simultaneously, the `plotEnrichment()` function can be used. Both the numbers of "is DA" and "is not DA" features are plotted in Figure 24. Their interpretation is quite straightforward: *is DA* are the positives, while the *is not DA* the negatives. Positives reported in Figure 24 are the True Positives, while negatives are the FPs.

```
plotEnrichment(enrichment = enrichment_nodir, enrichmentCol = "Reality")
```

From this example, only 9 out of 25 methods are able to find an enriched amount of truly DA features without any false discovery: `dearseq.counts.permutation.1000` in the first position. On the contrary, `basic.t.counts`, `DESeq2.counts.poscounts.weighted`, `limma.counts.TMM.weighted`, `metagenomeSeq.counts.CSS.fitFeatureModel`, `ALDEx2.counts.iqlr.wilcox.unpaired`, `MAST.counts.default`, `MAST.counts.median`, `ANCOM.counts`, and `NOISeq.counts.tmm` methods do not find any DA feature. This could be strongly related to the template taxa chosen to simulate the DA features.

To further assess methods' power, the `createPositives()` function can be used specifying as TPs the resulting DA features created as real DA features and as FPs the resulting DA features created as not DA features (Figure 25).

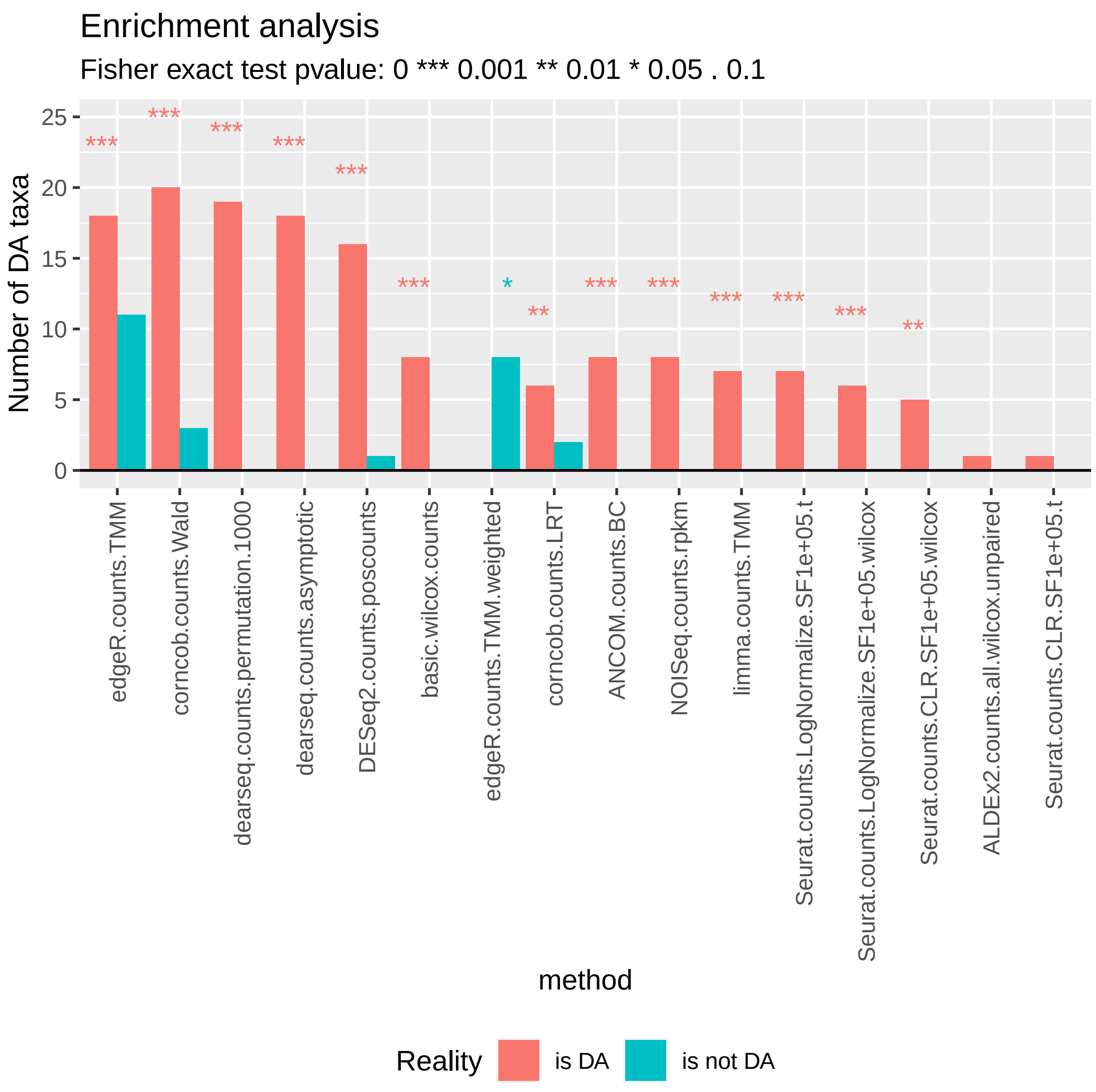

Figure 24: Enrichment plot for simulated data. Number of differentially abundant features, colored by DA reality.

We use a *threshold_pvalue = 0.1* (*0.4* for ANCOM) to call a feature DA based on its adjusted p-value. We compute the difference between TPs and FPs for several *top* thresholds (from 5 to 30, by 5) in order to observe a trend:

```
positives_nodir <- createPositives(
    object = sim_DA,
    priorKnowledge = priorInfo,
    enrichmentCol = "Reality",
    namesCol = NULL,
    slot = "pValMat",
    colName = "adjP",
    type = "pvalue",
    threshold_pvalue = c(
        rep(0.1,19), # adjP thresholds
        0.4, # adjP threshold for ANCOM on 1-W/(ntaxa-1)
        rep(0.1,5)), # adjP thresholds for other methods
    threshold_logfc = 0,
    top = seq(5, 30, by = 5),
    alternative = "greater",
    verbose = FALSE,
    TP = list(c("DA", "is DA")),
    FP = list(c("DA", "is not DA"))
)
```

Since the number of simulated DA feature is 20, the maximum number of TPs is 20 and it is added as an horizontal line to the figure.

```
plotPositives(positives = positives_nodir) +
    facet_wrap( ~ method) +
    theme(legend.position = "none") +
    geom_hline(aes(yintercept = 20), linetype = "dotted", color = "red") +
    geom_hline(aes(yintercept = 0), color = "black") +
    ylim(NA, 21)
```

From figure 25 it is clearly visible that `dearseq.counts.permutation.1000` and `dearseq.counts.asymptotic` reach the highest values of the difference, followed by `corncob.counts.Wald` and `DESeq2.counts.poscounts`. As already mentioned the desired level of power which a methods should be able to reach is represented by the red dotted line, *i.e.* the total number of DA simulated features (20 in our case). These methods, in this specific example, have the highest power. Differently, methods characterized by flat lines have a fixed number of features with an adjusted p-value lower than the threshold. If their lines are above the zero line, it means that the number of True Positives is greater than the number of FPs. On the contrary, if their lines are below the zero line, it means that the number of FPs is greater (*e.g.*, `edgeR.counts.TMM.weighted` has negative values, maybe due to poor weight estimates since all methods with observational weights perform poorly).

6.9 Discussion about Enrichment

The enrichment analysis toolbox provides many methods to study DA in a dataset.

Firstly, when some prior knowledge is available, it allows to evaluate methods' power. Among the possible uses, it is especially useful to investigate conservative methods: are they calling only the most obvious taxa (also found by the other methods) or are they finding something new? The main drawback is that the availability of the prior knowledge is limited, especially for new datasets. For this reason, enrichment analysis could also be used in addition to simulation tools. Indeed, through parametric, semi-parametric, or non parametric assumptions it is possible to obtain an emulation of the prior knowledge.

Secondly, thanks to methods like `plotMutualFindings()` and `plotEnrichment()`, which produce graphical results like Figure 22 and Figure 23, it is also possible to use the enrichment analysis to study the distribution

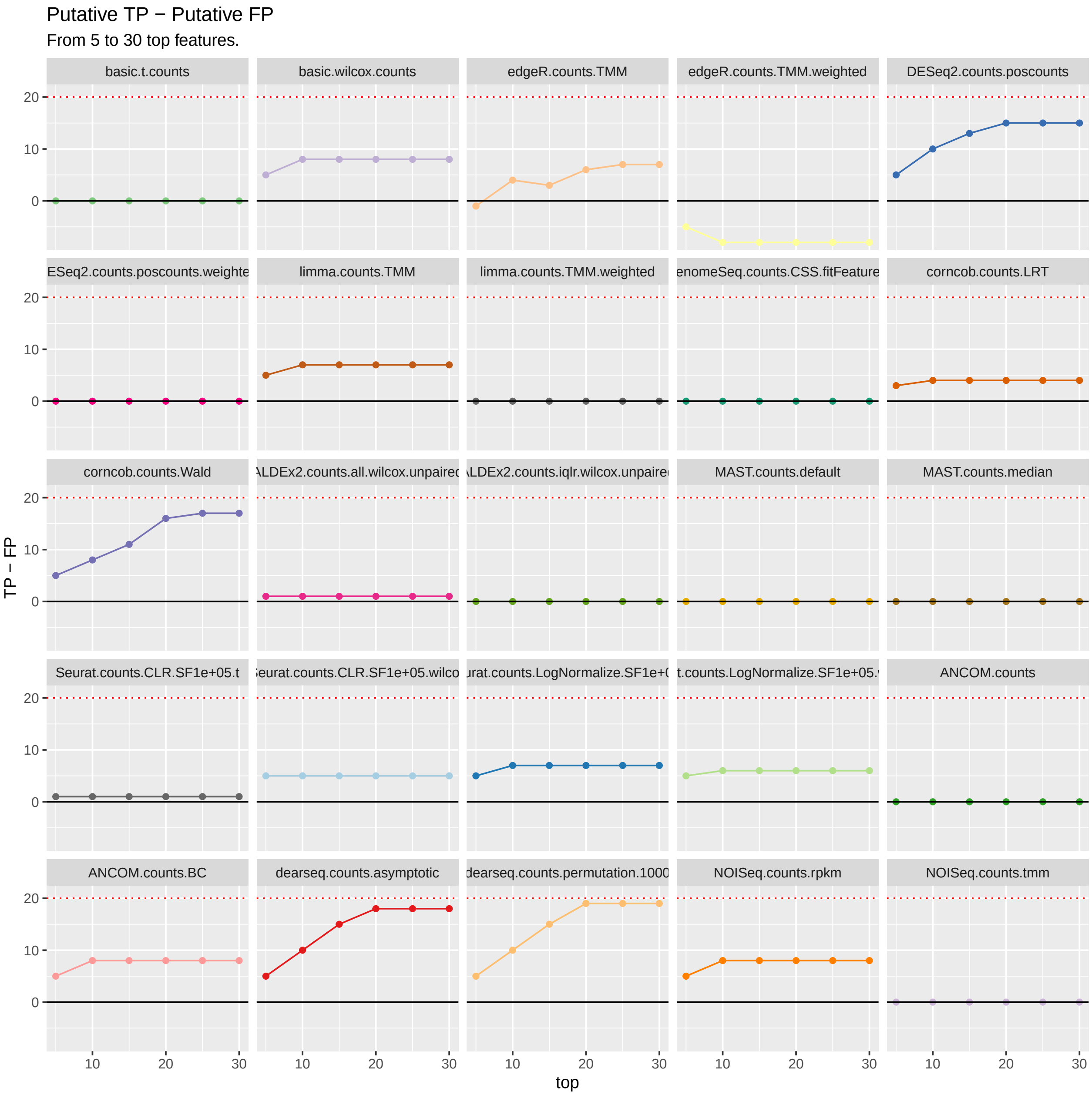

Figure 25: TP, FP differences plot. Differences between the number of True Positives and False Positives for several thresholds of the top ranked adjusted p-values lower than 0.1 (the top 5 lowest adjusted p-values, the top 10, 15, ..., 30) for each method in simulated data. Red dotted line represents the total number of DA simulated features.

of the findings across class of taxa (*e.g.*, by using as prior knowledge the phylum of the features, it would be possible to study if a phylum is characterized by an increased number of DA compared to another phylum), or more simply, drawing biological conclusions based only on taxa found as DA by the majority of the methods.

7 Session Info

```
sessionInfo()
```

```
## R version 4.2.1 (2022-06-23)
## Platform: x86_64-apple-darwin17.0 (64-bit)
## Running under: macOS Big Sur ... 10.16
##
## Matrix products: default
## BLAS:   /Library/Frameworks/R.framework/Versions/4.2/Resources/lib/libRblas.0.dylib
## LAPACK: /Library/Frameworks/R.framework/Versions/4.2/Resources/lib/libRlapack.dylib
##
## locale:
## [1] it_IT.UTF-8/it_IT.UTF-8/it_IT.UTF-8/C/it_IT.UTF-8/it_IT.UTF-8
##
## attached base packages:
## [1] stats4    stats     graphics  grDevices utils     datasets  methods
## [8] base
##
## other attached packages:
##  [1] kableExtra_1.3.4            cowplot_1.1.1
##  [3] ggplot2_3.4.0               plyr_1.8.7
##  [5] SummarizedExperiment_1.28.0 Biobase_2.58.0
##  [7] GenomicRanges_1.50.1        GenomeInfoDb_1.34.2
##  [9] IRanges_2.32.0              S4Vectors_0.36.0
## [11] BiocGenerics_0.44.0         MatrixGenerics_1.10.0
## [13] matrixStats_0.62.0          phyloseq_1.42.0
## [15] SPsimSeq_1.8.0              BiocParallel_1.32.1
## [17] benchdamic_1.5.1
##
## loaded via a namespace (and not attached):
##   [1] rsvd_1.0.5                      svglite_2.1.0
##   [3] zinbwave_1.20.0                 Hmisc_4.7-1
##   [5] ica_1.0-3                       class_7.3-20
##   [7] glmnet_4.1-4                    foreach_1.5.2
##   [9] lmtest_0.9-40                   crayon_1.5.2
##  [11] rbibutils_2.2.9                 MASS_7.3-58.1
##  [13] rhdf5filters_1.10.0             MAST_1.24.0
##  [15] nlme_3.1-160                    backports_1.4.1
##  [17] ALDEx2_1.30.0                   impute_1.72.0
##  [19] rlang_1.0.6                     XVector_0.38.0
##  [21] ROCR_1.0-11                     readxl_1.4.1
##  [23] irlba_2.3.5.1                   nloptr_2.0.3
##  [25] limma_3.54.0                    scater_1.26.0
##  [27] bit64_4.0.5                     glue_1.6.2
##  [29] rngtools_1.5.2                  sctransform_0.3.5
##  [31] parallel_4.2.1                  vipor_0.4.5
##  [33] spatstat.sparse_3.0-0           AnnotationDbi_1.60.0
##  [35] VGAM_1.1-7                      spatstat.geom_3.0-3
##  [37] tidyselect_1.2.0                SeuratObject_4.1.3
##  [39] NADA_1.6-1.1                    fitdistrplus_1.1-8
##  [41] XML_3.99-0.12                   tidyr_1.2.1
##  [43] zoo_1.8-11                      xtable_1.8-4
##  [45] magrittr_2.0.3                  evaluate_0.18
```

```
##  [47] Rdpack_2.4                     scuttle_1.8.0
##  [49] cli_3.4.1                      zlibbioc_1.44.0
##  [51] rstudioapi_0.14                doRNG_1.8.2
##  [53] miniUI_0.1.1.1                 sp_1.5-1
##  [55] MultiAssayExperiment_1.24.0    metagenomeSeq_1.40.0
##  [57] rpart_4.1.19                   zCompositions_1.4.0-1
##  [59] tidytext_0.3.4                 treeio_1.22.0
##  [61] shiny_1.7.3                    BiocSingular_1.14.0
##  [63] xfun_0.34                      multtest_2.54.0
##  [65] cluster_2.1.4                  caTools_1.18.2
##  [67] biomformat_1.26.0              KEGGREST_1.38.0
##  [69] tibble_3.1.8                   expm_0.999-6
##  [71] ggrepel_0.9.2                  ape_5.6-2
##  [73] listenv_0.8.0                  Biostrings_2.66.0
##  [75] png_0.1-7                      permute_0.9-7
##  [77] future_1.29.0                  withr_2.5.0
##  [79] slam_0.1-50                    bitops_1.0-7
##  [81] cellranger_1.1.0               RcppZiggurat_0.1.6
##  [83] e1071_1.7-12                   survey_4.1-1
##  [85] pillar_1.8.1                   gplots_3.1.3
##  [87] cachem_1.0.6                   Rmpfr_0.8-9
##  [89] multcomp_1.4-20                DelayedMatrixStats_1.20.0
##  [91] vctrs_0.5.0                    ellipsis_0.3.2
##  [93] generics_0.1.3                 ROI.plugin.lpsolve_1.0-1
##  [95] tools_4.2.1                    foreign_0.8-83
##  [97] NOISeq_2.42.0                  beeswarm_0.4.0
##  [99] munsell_0.5.0                  emmeans_1.8.2
## [101] proxy_0.4-27                   DelayedArray_0.24.0
## [103] fastmap_1.1.0                  compiler_4.2.1
## [105] abind_1.4-5                    httpuv_1.6.6
## [107] DescTools_0.99.47              plotly_4.10.1
## [109] decontam_1.18.0                GenomeInfoDbData_1.2.9
## [111] gridExtra_2.3                  edgeR_3.40.0
## [113] lattice_0.20-45                deldir_1.0-6
## [115] utf8_1.2.2                     later_1.3.0
## [117] dplyr_1.0.10                   jsonlite_1.8.3
## [119] tokenizers_0.2.3               scales_1.2.1
## [121] gld_2.6.6                      ScaledMatrix_1.6.0
## [123] tidytree_0.4.1                 pbapply_1.5-0
## [125] sparseMatrixStats_1.10.0       estimability_1.4.1
## [127] genefilter_1.80.0              lazyeval_0.2.2
## [129] promises_1.2.0.1               doParallel_1.0.17
## [131] latticeExtra_0.6-30            goftest_1.2-3
## [133] spatstat.utils_3.0-1           reticulate_1.26
## [135] checkmate_2.1.0                rmarkdown_2.18
## [137] sandwich_3.0-2                 webshot_0.5.4
## [139] statmod_1.4.37                 Rtsne_0.16
## [141] softImpute_1.4-1               uwot_0.1.14
## [143] igraph_1.3.5                   survival_3.4-0
## [145] numDeriv_2016.8-1.1            yaml_2.3.6
## [147] systemfonts_1.0.4              ANCOMBC_2.0.1
## [149] htmltools_0.5.3                memoise_2.0.1
## [151] Seurat_4.2.1                   locfit_1.5-9.6
## [153] viridisLite_0.4.1              gmp_0.6-7
```

```
## [155] digest_0.6.30                    assertthat_0.2.1
## [157] mime_0.12                        registry_0.5-1
## [159] RSQLite_2.2.18                   Rfast_2.0.6
## [161] yulab.utils_0.0.5                future.apply_1.10.0
## [163] Exact_3.2                        data.table_1.14.4
## [165] blob_1.2.3                       vegan_2.6-4
## [167] preprocessCore_1.60.0            detectseparation_0.3
## [169] lpSolveAPI_5.5.2.0-17.9          labeling_0.4.2
## [171] splines_4.2.1                    Formula_1.2-4
## [173] DECIPHER_2.26.0                  Rhdf5lib_1.20.0
## [175] RCurl_1.98-1.9                   hms_1.1.2
## [177] rhdf5_2.42.0                     colorspace_2.0-3
## [179] base64enc_0.1-3                  shape_1.4.6
## [181] ggbeeswarm_0.6.0                 nnet_7.3-18
## [183] TreeSummarizedExperiment_2.6.0 mia_1.6.0
## [185] Rcpp_1.0.9                       RANN_2.6.1
## [187] mvtnorm_1.1-3                    fansi_1.0.3
## [189] SnowballC_0.7.0                  truncnorm_1.0-8
## [191] parallelly_1.32.1                R6_2.5.1
## [193] grid_4.2.1                       ggridges_0.5.4
## [195] lifecycle_1.0.3                  rootSolve_1.8.2.3
## [197] minqa_1.2.5                      leiden_0.4.3
## [199] dearseq_1.10.0                   fastcluster_1.2.3
## [201] Matrix_1.5-1                     corncob_0.3.0
## [203] RcppAnnoy_0.0.20                 TH.data_1.1-1
## [205] RColorBrewer_1.1-3               iterators_1.0.14
## [207] spatstat.explore_3.0-3           stringr_1.4.1
## [209] htmlwidgets_1.5.4                Wrench_1.16.0
## [211] beachmat_2.14.0                  polyclip_1.10-4
## [213] purrr_0.3.5                      ROI_1.0-0
## [215] janeaustenr_1.0.0                rvest_1.0.3
## [217] mgcv_1.8-41                      CVXR_1.0-11
## [219] globals_0.16.1                   lmom_2.9
## [221] htmlTable_2.4.1                  patchwork_1.1.2
## [223] spatstat.random_3.0-1            progressr_0.11.0
## [225] codetools_0.2-18                 GO.db_3.16.0
## [227] prettyunits_1.1.1                gtools_3.9.3
## [229] SingleCellExperiment_1.20.0      gtable_0.3.1
## [231] DBI_1.1.3                        dynamicTreeCut_1.63-1
## [233] highr_0.9                        tensor_1.5
## [235] httr_1.4.4                       KernSmooth_2.23-20
## [237] progress_1.2.2                   stringi_1.7.8
## [239] farver_2.1.1                     reshape2_1.4.4
## [241] annotate_1.76.0                  viridis_0.6.2
## [243] xml2_1.3.3                       ggdendro_0.1.23
## [245] trust_0.1-8                      boot_1.3-28
## [247] WGCNA_1.71                       BiocNeighbors_1.16.0
## [249] lme4_1.1-31                      interp_1.1-3
## [251] ade4_1.7-20                      geneplotter_1.76.0
## [253] CompQuadForm_1.4.3               energy_1.7-10
## [255] scattermore_0.8                  DESeq2_1.38.0
## [257] bit_4.0.4                        jpeg_0.1-9
## [259] spatstat.data_3.0-0              pkgconfig_2.0.3
## [261] lmerTest_3.1-3                   gsl_2.1-7.1
```

```
## [263] DirichletMultinomial_1.40.0    MGLM_0.2.1
## [265] mitools_2.4                    knitr_1.40
```

References

Beghini,F. *et al.* (2019) Tobacco exposure associated with oral microbiota oxygen utilization in the New York City Health and Nutrition Examination Study. *Ann Epidemiol*, **34**, 18–25.e3.

Butler,A. *et al.* (2018) Integrating single-cell transcriptomic data across different conditions, technologies, and species. *Nat. Biotechnol.*, **36**, 411–420.

Calgaro,M. *et al.* (2020) Assessment of statistical methods from single cell, bulk RNA-seq, and metagenomics applied to microbiome data. *Genome Biology*, **21**, 191.

Fernandes,A.D. *et al.* (2014) Unifying the analysis of high-throughput sequencing datasets: characterizing RNA-seq, 16S rRNA gene sequencing and selective growth experiments by compositional data analysis. *Microbiome*, **2**, 15.

Finak,G. *et al.* (2015) MAST: a flexible statistical framework for assessing transcriptional changes and characterizing heterogeneity in single-cell RNA sequencing data. *Genome Biol*, **16**, 278.

Gauthier,M. *et al.* (2020) dearseq: a variance component score test for RNA-seq differential analysis that effectively controls the false discovery rate. *NAR Genomics and Bioinformatics*, **2**, lqaa093.

Kaul,A. *et al.* (2017) Analysis of Microbiome Data in the Presence of Excess Zeros. *Front. Microbiol.*, **8**, 2114.

Law,C.W. *et al.* (2014) voom: Precision weights unlock linear model analysis tools for RNA-seq read counts. *Genome Biol.*, **15**, R29.

Lin,H. and Peddada,S.D. (2020) Analysis of compositions of microbiomes with bias correction. *Nat Commun*, **11**, 3514.

Love,M.I. *et al.* (2014) Moderated estimation of fold change and dispersion for RNA-seq data with DESeq2. *Genome Biol.*, **15**, 550.

Martin,B.D. *et al.* (2020) Modeling microbial abundances and dysbiosis with beta-binomial regression. *Ann. Appl. Stat.*, **14**.

Paulson,J.N. *et al.* (2013) Differential abundance analysis for microbial marker-gene surveys. *Nat. Methods*, **10**, 1200–1202.

Phipson,B. *et al.* (2016) Robust hyperparameter estimation protects against hypervariable genes and improves power to detect differential expression. *Ann. Appl. Stat.*, **10**.

Risso,D. *et al.* (2018) A general and flexible method for signal extraction from single-cell RNA-seq data. *Nat. Commun.*, **9**, 284.

Ritchie,M.E. *et al.* (2015) limma powers differential expression analyses for RNA-sequencing and microarray studies. *Nucleic Acids Research*, **43**, e47–e47.

Robinson,M.D. *et al.* (2010) edgeR: a Bioconductor package for differential expression analysis of digital gene expression data. *Bioinformatics*, **26**, 139–140.

Schiffer,L. *et al.* (2019) HMP16SData: Efficient Access to the Human Microbiome Project Through Bioconductor. *Am. J. Epidemiol.*, **188**, 1023–1026.

Tarazona,S. *et al.* (2015) Data quality aware analysis of differential expression in RNA-seq with NOISeq R/Bioc package. *Nucleic Acids Res*, gkv711.

Thurnheer,T. *et al.* (2016) Microbial dynamics during conversion from supragingival to subgingival biofilms in an in vitro model. *Molecular Oral Microbiology*, **31**, 125–135.

Van den Berge,K. *et al.* (2018) Observation weights unlock bulk RNA-seq tools for zero inflation and single-cell applications. *Genome Biol.*, **19**, 24.

Zhang,Y. *et al.* (2017) Regression Models for Multivariate Count Data. *Journal of Computational and Graphical Statistics*, **26**, 1–13.

Zhang,Y. and Zhou,H. (2022) MGLM: Multivariate Response Generalized Linear Models.
